# A Crosstalk- and Interferent-Free Dual Electrode Amperometric Biosensor for the Simultaneous Determination of Choline and Phosphocholine

**DOI:** 10.3390/s21103545

**Published:** 2021-05-19

**Authors:** Rosanna Ciriello, Antonio Guerrieri

**Affiliations:** Dipartimento di Scienze, Università degli Studi della Basilicata, Viale dell’Ateneo Lucano 10, 85100 Potenza, Italy; rosanna.ciriello@unibas.it

**Keywords:** choline analysis, phosphocholine analysis, choline oxidase, alkaline phosphatase, enzyme immobilization, overoxidized polypyrrole, electropolymerized non-conducting polymer, dual electrode biosensor, simultaneous determination, flow injection analysis

## Abstract

Choline (Ch) and phosphocholine (PCh) levels in tissues are associated to tissue growth and so to carcinogenesis. Till now, only highly sophisticated and expensive techniques like those based on NMR spectroscopy or GC/LC- high resolution mass spectrometry permitted Ch and PCh analysis but very few of them were capable of a simultaneous determination of these analytes. Thus, a never reported before amperometric biosensor for PCh analysis based on choline oxidase and alkaline phosphatase co-immobilized onto a Pt electrode by co-crosslinking has been developed. Coupling the developed biosensor with a parallel sensor but specific to Ch, a crosstalk-free dual electrode biosensor was also developed, permitting the simultaneous determination of Ch and PCh in flow injection analysis. This novel sensing device performed remarkably in terms of sensitivity, linear range, and limit of detection so to exceed in most cases the more complex analytical instrumentations. Further, electrode modification by overoxidized polypyrrole permitted the development of a fouling- and interferent-free dual electrode biosensor which appeared promising for the simultaneous determination of Ch and PCh in a real sample.

## 1. Introduction

Choline (Ch), a vitamin-like nutrient found in many common foods, is essential for several biological functions in human body [1]. Three different pathways are involved in its In Vivo metabolism [1]. Its phosphorylation by choline kinase, an enzyme widely distributed in mammalian tissues, gives phosphocholine (PCh), the precursor involved in the Kennedy pathway for the biosynthesis of phosphatidylcholine. On the other hand, mainly in liver and kidney, Ch is irreversibly oxidized to betaine, a methyl donor involved in the biosynthesis of methionine. Last, but not less important, Ch is acetylated, essentially in cholinergic neurons, by acetylcholine transferase to acetylcholine (ACh), an important and well-known neurotransmitter.

The Ch uptake and its metabolism are tissue-dependent so the levels of Ch and its metabolites are related to tissue growth and plausibly to carcinogenesis [2,3]. In fact, Ch levels change significantly in malignant tissue transformations [3] while abnormal PCh levels were found in many human cancers and transformed cell lines [2]; even several chemicals, well-known to be carcinogenic, appear to stimulate the formation of PCh in several cell types [2]. As a proof of these findings, abnormal levels of Ch and PCh were found in some human tumors [4], mainly in breast cancers [3,5,6,7]; in addition, some studies pointed out similar features also for non-Hodgkin’s lymphoma [8] and endometrial cancers [9]. Too, abnormal levels were also observed for other diseases like chronic pancreatitis [10], polycystic ovary syndrome [11], and some serious neurological diseases [12,13], thus pointing out the relevance of Ch and PCh levels in clinical diagnostics. Remarkably, Ch and PCh levels are related due to the Kennedy pathway, so often the ratio of Ch/PCh levels, rather than their absolute levels, is considered as a diagnostic and prognostic marker.

While many analytical methods have been developed for Ch analysis, few approaches were dedicated to PCh and even fewer for the simultaneous determination of both analytes. Pomfret et al. [14] described a procedure able to isolate and detect Ch and PCh in biological tissues and fluids. Unfortunately, the approach appears quite complex, laborious, and time consuming since requiring a preliminary HPLC separation of analytes, further isolation by TLC, chemical conversion of PCh in Ch for at least 24 h, and final quantitation by GC-MS after Ch derivatization. Koc et al. [15] circumvented all these separation procedures by using a LC separation coupled to electrospray ionization—isotope dilution mass spectrometry (LC/ESI-IDMS). This method permitted the analysis of Ch, PCh, and other related compounds in e.g., mouse liver and rat fetal brain, but required the use of deuterium-labeled internal standards of all the analytes, lengthy time analysis, and much more expensive equipment. Bioimaging of PCh, cholesterol, and galactosylceramide on rat cerebellar cortex was possible by time-of-flight secondary-ion-mass-spectrometry (TOF-SIMS) [16], but unfortunately, simultaneous detection of Ch was not reported. A hydrophilic interaction liquid chromatography combined with high resolution ESI mass spectrometry (HILIC LC-MS/MS) method has been proposed for the analysis of Ch, PCh, and other related Ch compounds in egg yolks [17] and rat livers [18]. Nevertheless, this approach requires the use of deuterated standards and a complex as expensive equipment like QTRAP™ MS which heavily limit its use in clinical laboratories; fortunately, Mimmi et al. [19,20] showed that the same approach could be pursued with less MS demand, but 1D NMR was required as a complementary analytical technique. Finally, an ultrahigh performance liquid chromatography (UPLC)—tandem mass spectroscopy was developed for the simultaneous analysis of Ch, PCh, and other involved neurotransmitters and metabolites [21] but this approach was validated only for zebrafish embryos and larvae.

Certainly, NMR spectroscopy represents today one of the most used approach for human cancer characterization, diagnostic and prognostic analysis in clinical environment [4] and hence for the simultaneous analysis of Ch and PCh in those tissues. Further, 1D and 2D proton NMR has been used for the biochemical characterization of perchloric acid extracts of metastatic lymph nodes of breast cancer patients [5]. ^31^ P edited ^1^ H NMR spectroscopy permitted quantification of Ch, PCh, and other analytes in human brain tumor extracts [22]. ^1^ H high resolution magic angle spinning NMR spectroscopy allowed metabolic profiling of Ch and PCh in human lung cancer tissues [23]. High field (7T) ^31^ P NMR imaging coupled to ^1^ H NMR spectroscopy demonstrated useful for the non-invasive determination of important biomarkers in human breast [7] and prostate [24] cancers but determination of Ch and PCh required two techniques and cannot be considered a simultaneous determination. ^1^ H high resolution NMR spectroscopy was recently described as able to analyze Ch and PCh in fine needle aspirate biopsies of breast cancer lesions [25]. In Vivo ^31^ P NMR imaging allowed metabolic profiling of human breast cancer xenografts [26] but Ch analysis was not possible. NMR approaches were also described for the analysis of Ch and PCh in solutions [27], in liver tissues [28], as well as in ovarian cancer diagnostic [29] but this latter required peculiar signal data processing [30,31] instead of the conventional Fourier transform usually found in commercial NMR data stations.

Even if representing the best actual approach in clinical studies and analysis, NMR spectroscopy is not without drawbacks when applied for Ch and PCh simultaneous analysis. As an example, water-suppressed ^1^ H NMR spectroscopy is required for total choline containing compounds analysis [4]. In addition, the water signal is used as an internal reference for the absolute quantification of metabolites so additional measurements are required for the acquisition of this internal reference [32]. Further, ^1^ H NMR spectroscopy is not able to resolve Ch and PCh signals as well as those between PCh and related phospholipid compounds [4,7] even in simple biological fluids like urine [33] so that the use of ^13^ C labeling is required [3]; a similar failure was observed also for ^31^P NMR when decoupling of PCh and phosphoethanolamine signals is required [4]. Even in the most capable approaches (as above described), finding or using a “blank” tissue (i.e., tissues with cells which are the normal counterparts) for comparing cancer spectra is not straightforward [4]: this is possible for breast, primary liver, prostate, and glioma cancers but not for sarcomas, lymphomas, melanomas, and head or neck carcinomas where the background contains skeletal muscle, which of course cannot be considered the normal counterpart; moreover, for primary brain tumors, where the single voxel localization technique is used to circumvent interferent signals from normal brain parts, the tumor is usually surrounded by edema, so in NMR scans, it is difficult to establish where the cancer ends and the edema begins [4].

As surely it is evident, the above approaches, whatever well-established in some cases, all show a restricted or at least complicated practicability since requiring complex and expensive instruments and thus skilled operators or/and time-consuming and complicate sample pre-treatments, e.g., do not satisfy the requirements for a simple, fast, specific, and accurate analysis, usually necessary in clinical and medical fields. In this respect, immobilized enzyme sensors, i.e., biosensors, can play undoubtedly an important role since compacts, easy in their use, almost cheap and producing analytical responses quickly, with high sensitivity and specificity, thus not requiring any sample pre-treatment. Unfortunately, enzymatic approaches for Ch and PCh simultaneous analysis are still not described till now. Masoom et al. [34] described a method based on immobilized enzyme reactors (IMERs) and amperometric detection of the released hydrogen peroxide according to the following enzymatic reactions with both analytes:

$$phosphocholine+H_{2}O\;\frac{ALP}{\;}>choline+phosphate\;choline+2O_{2}+H_{2}O\;\frac{ChO}{\;}>betaine+2H_{2}O_{2}$$

Accordingly, using a choline oxidase (ChO)-based IMER or an alkaline phosphatase (ALP)-based IMER coupled in series with a ChO-IMER, they were able to analyze Ch and PCh, respectively, but a simultaneous determination of both analytes was not reported. An HPLC procedure coupled to an acetylcholine esterase/ChO-IMER followed by amperometric detection permitted the analysis of Ch and its metabolites in rat brain and body fluids [35] as well as in mouse tissue [36]. Nevertheless, PCh analysis necessitated sample pre-treatment and incubation with ALP before analysis, whereas its quantification came from the increase of free Ch peak after ALP hydrolysis so that the simultaneous determination of both analytes was unachievable.

In the field of Ch biosensing, we have already described a fast response and sensitive amperometric biosensor for Ch and ACh analysis [37]. Based on ChO and acetylcholine esterase (AChE) immobilized onto a Pt electrode through co-crosslinking with bovine serum albumin by glutaraldehyde, the immobilization procedure there developed proved rapid, easy but robust and simply adaptable for any working electrode configuration, even for conventional HPLC flow cells, thus permitting, e.g., the development of a novel HPLC detector for Ch and ACh determination in brain tissue homogenates [38]. Further, this latter can easily be modifiable in dual electrode configuration, thus permitting, after proper anti-fouling and anti-interference electrode modification (see below), the simultaneous detection of both analytes without the need of any chromatographic separation [39]. The developed biosensor proved even useful for the realization of a disposable device aimed at a rapid screening of AChE activity in soil extract [40] or to the development of novel clinical assays for serum cholinesterase activity [41] or Ch detection in biological fluids from patients on hemodialysis [42].

Therefore, in the present paper, it will be described for the first time the development of a novel amperometric biosensor for PCh analysis based on ALP and ChO co-immobilized onto a Pt electrode by co-crosslinking; more importantly, it will be demonstrated that a simultaneous determination of Ch and PCh can be achieved by a crosstalk-free dual electrode amperometric biosensor based on ChO and ChO-ALP enzyme immobilized onto Pt electrodes by co-crosslinking. Electropolymerized non-conducting films with built-in permselectivity [39,41,42,43,44,45,46,47,48,49,50,51,52,53,54] have widely demonstrated to be able, in real sample analysis (e.g., blood, serum), to protect the sensor surface from electrode fouling effects and to remove, with unmatched efficiency, common interfering species in glucose, Ch, and Ach, as well as in lysine determination. Accordingly, electrode modification by overoxidized polypyrrole permitted us to develop a fouling- and interferent-free dual electrode biosensor which appeared promising for the simultaneous determination of Ch and PCh in the clinical sample.

## 2. Materials and Methods

### 2.1. Materials

Choline chloride, phosphocholine chloride calcium salt tetrahydrate, choline oxidase (EC 1.1.3.17 from *Alcaligenes species*, 14 U/mg of solid), alkaline phosphatase (EC 3.1.3.1 from bovine intestinal mucosa, 13 DEA units/mg of solid, product code P7640), alkaline phosphatase (EC 3.1.3.1 from bovine calf intestinal, 2,000 DEA units, product code P7923), alkaline phosphatase (EC 3.1.3.1 from shrimp, 1,300 DEA units, product code P9088), ascorbic acid, *L*-cysteine, glutaraldehyde (grade II, 25% aqueous solution), and bovine serum albumin (fraction V) were bought from Sigma Chemical Co. (St. Louis, MO, USA). Alkaline phosphatase (EC 3.1.3.1 from bovine intestinal mucosa, 22,990 U/mL, product code 79835), uric acid, and pyrrole came from Aldrich (Steinheim, Germany). Analytical reagent grade chemicals were used as other materials. Double distilled-deionized water was used to prepare all solutions. Choline chloride was dried under vacuum over P_2_O_5_ for at least 3 days and stored in a vacuum desiccator at 4 °C. When required, phosphocholine was purified by using a Strata-X-C 33 µm polymeric strong cation exchange tube (Phenomenex, Castel Maggiore (BO), Italy). Pyrrole was purified by vacuum distillation (62 °C) and stored at 4 °C. Choline and phosphocholine stock solutions were stored at 4 °C: their dilute solutions, pyrrole and interferant solutions were prepared just before their use.

### 2.2. Apparatus

Batch electrochemical, rotating disk electrode (RDE) experiments were performed using an AMEL (Milan, Italy) Model 466 polarographic analyzer coupled to a JJ instruments mod. CR65OS Yt chart recorder. The electrochemical cell was a conventional three-electrode system consisting of an Ag/AgCl, KCl satd. reference electrode, a Pt counter electrode, and a Pt RDE. The RDE was a CTV101 Speed Control Unit, EDI 101 Rotating Disc Electrode (Radiometer, Copenhagen, Denmark).

Flow injection experiments were performed using a Gilson Minipuls 3 peristaltic pump (Gilson Medical Electronics, Villiers-Le-Bel, France) and a seven-port injection valve (Rheodyne mod. 7725, Cotati, CA, USA) with a 20 µL sample loop. The electrochemical detector was an EG&G Model 400 (Princeton Applied Research, Princenton, NJ, USA) including a thin-layer electrochemical cell with a single Pt disk (3 mm diameter) working electrode and an Ag/AgCl, 3 M NaCl reference electrode; for dual electrodes experiments, a dual Pt disk (both 3 mm diameter) working electrode (parallel configuration) was used; in both cases, two thin layer flow cell dual gaskets (Bioanalytical Systems, West Lafayett, IN, USA) of 0.004-inch thickness were used. A PEEK tubing (0.25 mm ID, 150 cm length) was used to connect the sample injection valve to the electrochemical detector. A Kipp & Zonen (Delft, The Netherlands) mod. BD 12 Flatbed recorder was used for flow injection signal chart recording. The flow injection setup is illustrated in Scheme S1 in Supplementary Materials.

Controlled electrochemical deposition of polypyrrole film onto the single or dual Pt electrodes was carried on a conventional three electrode cell equipped with an Ag/AgCl, KCl satd. reference electrode and a Pt counter electrode by using an EG&G model 263A potentiostat/galvanostat equipped with a M270 electrochemical research software (EG&G Princeton Applied Research, Princeton, NJ, USA) version 4.23 for data control and acquisition.

### 2.3. Methods

#### 2.3.1. Electrode Modification

Before each electrode modification, the Pt disk working electrode of the thin-layer electrochemical cell was preliminary cleaned by few drops of hot nitric acid, washed with bidistilled water, then polished to a mirror finish by alumina (0.05 µm particles), and finally extensive washed and sonicated in bidistilled water. To check the presence of electrode surface impurities as well as for electrode conditioning, the electrodes were cycled in 0.5 M sulfuric acid solution between −0.210 and + 1.190 V (vs. Ag/AgCl, KCl satd.) at 200 mV/s until a steady state cyclic voltammogram was obtained, followed by copious rinsing with double distilled-deionized water.

Platinum disk working electrodes were modified by electrosynthesized polypyrrole (PPy) films by potentiostatically growth at + 0.7 V (vs. Ag/AgCl, KCl satd.) from a 0.1 M KCl supporting electrolyte solution containing 0.4 M pyrrole; a deposition charge of 300 mC/cm^2^ was typically used permitting the growth of an approximately 0.7 µm thick film [55]. The so-obtained Pt/PPy modified electrodes were successively overoxidized at + 0.7 V (vs. Ag/AgCl, KCl satd.) in phosphate buffer (pH 7, *I* 0.1 M) for about 6 h until a steady-state background current was observed; the resultant overoxidized Pt/PPy electrodes (Pt/oPPy) were then washed with double distilled-deionized water and air-dried at room temperature.

#### 2.3.2. Biosensor Preparation

Choline oxidase (ChO) was immobilized onto bare Pt or Pt/oPPy electrodes as follow. Then, 1.00 mg of ChO (corresponding to 14 units) and 16 mg of bovine serum albumin (BSA) were carefully dissolved avoiding air bubble formation into 200 µL of phosphate buffer (pH 6.5, *I* 0.1 M). The resulting solution was divided in two identical aliquots of 100 µL. An aliquot was mixed with 50 µL of phosphate buffer (pH 6.5, *I* 0.1 M) and 15 µL of 2.5% glutaraldehyde (GLU) solution (25% GLU solution diluted 1:10 with phosphate buffer pH 7.4, *I* 0.1 M) and used for ChO biosensor preparation. Alkaline phosphatase (ALP) and ChO were co-immobilized onto bare Pt or Pt/oPPy electrodes as follow. The remaining aliquot of the previous prepared ChO-BSA solution was mixed with 50 µL of ALP (22,990 U/mL) and 15 µL of 2.5% GLU solution and used for ChO-ALP biosensor preparation.

ChO and ChO-ALP biosensors were prepared as follow. Three µL of the enzyme solution from the appropriate aliquots described above were carefully pipetted onto the bare Pt or Pt/oPPy disk working electrode surface and meticulously spread out to cover completely the electrode surface avoiding air bubble formation. After that, the so modified electrode was left to crosslinking and air-drying at room temperature for few minutes. To avoid any cross-contamination from weakly bounded ALP onto ChO biosensor in dual electrodes experiments, both electrodes were successively cured with 10 µL of BSA solution (16 mg of BSA into 300 µL of phosphate buffer pH 6.5, *I* 0.1 M) to saturate any free glutaraldehyde residues left onto the enzyme membrane. Before their first use, the biosensors were preliminarily soaked in the background electrolyte for few minutes to allow removing of weakly bound or adsorbed enzyme and swelling of the enzyme layer. When not in use, the biosensor was stored in phosphate buffer, pH 6.5, *I* 0.1 M, at 4 °C in the dark.

#### 2.3.3. Electrochemical Measurements

The detection potential in all the electrochemical measurements was chosen as the lowest potential value, showing maximum sensitivity and pH independence towards hydrogen peroxide oxidation and, hence, towards analyte detection.

The detection potential in batch electrochemical, RDE experiments was + 0.7 V (vs. Ag/AgCl, KCl satd.) and the rotation rate was 1000 rpm. A borate buffer (pH 9.0, *I* 0.1 M) was used as supporting electrolyte and for sample analysis. Solutions were air saturated and the temperature was ambient.

In flow injection electrochemical measurements, the detection potential was +0.7 V (vs. Ag/AgCl, 3 M NaCl). Unless otherwise specified, in flow injection experiments, the carrier stream was a borate buffer (pH 9.0, *I* 0.1 M), the sample injection volume was 20 µL, and flow rate was 1 mL/min; solutions and carrier stream were air saturated and the temperature was ambient.

## 3. Results and Discussion

### 3.1. Enzyme Immobilization and Choice of ALP Source

Choline oxidase (ChO) was immobilized and ChO and alkaline phosphatase (ALP) were co-immobilized onto bare Pt or modified Pt electrodes by co-crosslinking [56], i.e., by crosslinking with glutaraldehyde (GLU) through the use of an inert protein, bovine serum albumin (BSA), sweetening the crosslinker reactivity. This valuable immobilization approach allowed us to obtain an immobilized enzyme layer with high biocomponent activity and good mechanical properties by simply casting a small amount of the proper co-crosslinking enzyme solution onto the electrode surface [37]. Obviously, the pH of enzyme immobilization solution and the inert protein and crosslinker concentrations have strong impact on the efficiency of enzyme immobilization and their catalytic properties so that their influence was studied and optimized. In agreement with a previous finding [37], the use of a phosphate buffer (pH 6.5, *I* 0.1 M) assured a good catalytic and mechanical stability of the immobilized enzyme layer while reducing the co-crosslinking reaction time; similarly, a BSA concentration of about 50 mg/mL and a GLU concentration of nearly 0.2% permitted high enzyme loadings, reasonable gelation times while reducing undesired denaturation effects. Finally, a volume *V*_c_ of 3 µL of the enzyme solution cast onto the electrode surface assured the formation of a strongly adherent, thin enzymatic membrane on the top of the working electrode with high permeation and low response time towards both analytes and the produced hydrogen peroxide (for a deepening about the influence of pH, BSA, GLU concentrations, and *V*_c_, as well as for a morphological study see references [37,42,53]).

In agreement with previous findings [37,38,39,40,41,42,54], ChO from *Alcaligenes species* (the almost unique available commercial source) proved successful for the realization of Ch biosensors. ALP, vice versa, comes from different sources and distinctive commercial preparations (see Section 2) so the choice of a satisfactory ALP source for successful PCh biosensing required the preparation of different Pt/ChO-ALP biosensors at different ratios of their enzyme activities and their preliminary analytical characterization in batch electrochemical, rotating disk electrode (RDE) experiments. In this context, absolute and relative analyte sensitivities, response time, operational and long-term stability of the relevant biosensors were the analytical performances here studied to select the optimal enzyme source (see Table S1 in Supplementary Materials for a relevant survey about). Almost all the enzyme sources gave biosensors with low absolute and relative PCh sensitivity respect to Ch one. Interesting, increasing the ALP to ChO activity ratio to increase the relative PCh sensitivity towards Ch did not give the expected result since the PCh absolute sensitivity remained almost the same. This suggested that the PCh response poorly depends on ChO loading (i.e., it is saturating) while Ch response is ChO loading limiting. Accordingly, tweaking the ALP to ChO activity ratio was unsuccessful for optimizing the performances of most biosensors. Anyway, among the different enzyme sources, ALP from bovine intestinal mucosa (22,990 U/mL, product code 79835, see Section 2) permitted the fabrication of Pt/ChO-ALP biosensors with the highest Ch and PCh absolute sensitivities, suitable sensitivity ratio, and high operational and long-term stability; notably, Pt/ChO-ALP biosensors as here developed showed enzyme layers so stable under stirred or flowing solutions and response times (as evaluated in batch RDE experiments) so low for both analytes (about 1 s) to allow their use in flow injection analysis without any distortion of the relevant flow injection sample peaks. Accordingly, this enzyme source was selected for further experiments where ChO and ALP were co-immobilized onto the Pt working electrode of a typical thin-layer electrochemical cell (see Scheme S1 in Supplementary Materials for further details).

### 3.2. Influence of Buffer Composition and Its pH

Preliminary, the effect of buffer composition was studied by using different buffers. In agreement with previous findings [54], phosphate and borate buffers showed the highest responses towards Ch at Pt/ChO-ALP electrodes; anyway, the PCh used in our experiments comes from its calcium salt (see Section 2), so phosphate buffers were excluded to avoid any calcium phosphate precipitation in solution.

The influence of pH on the Pt/Cho-ALP biosensor responses towards Ch and PCh was investigated in the pH range 6–10 using a universal buffer (acetate/borate/tris) at fixed ionic strength (*I* 0.1 M) as supporting electrolyte (note that the influence of pH on Pt/ChO biosensor was studied and reported elsewhere [37]). As Figure S1 in Supplementary Materials shows, a characteristic bell-shaped curve was observed for both analytes with a maximum located at about pH 9; notably, the behavior observed at the Pt/Cho-ALP biosensor towards Ch agrees with that observed at a Pt/ChO biosensor [37], suggesting that the immobilized ALP does not induce any significant change on ChO kinetics. Further, the observed maximum for immobilized ALP agrees with the optimal pH reported for its soluble form [57,58], indicating that the enzyme membrane does not influence significantly the ionization process of the immobilized enzyme.

Finally, the effect of magnesium ions on PCh response at the Pt/Cho-ALP biosensor was also investigated since an enhancement of ALP activity by Mg^++^ was reported elsewhere [59]. On contrast, in the present case, no PCh response increase was observed using magnesium chloride up to 0.5 mM so this salt was not used in the supporting electrolyte.

Accordingly, a borate buffer at pH 9 was used in all the experiments.

### 3.3. Analytical Performances of the Dual Electrode Biosensor

**Scheme 1:**
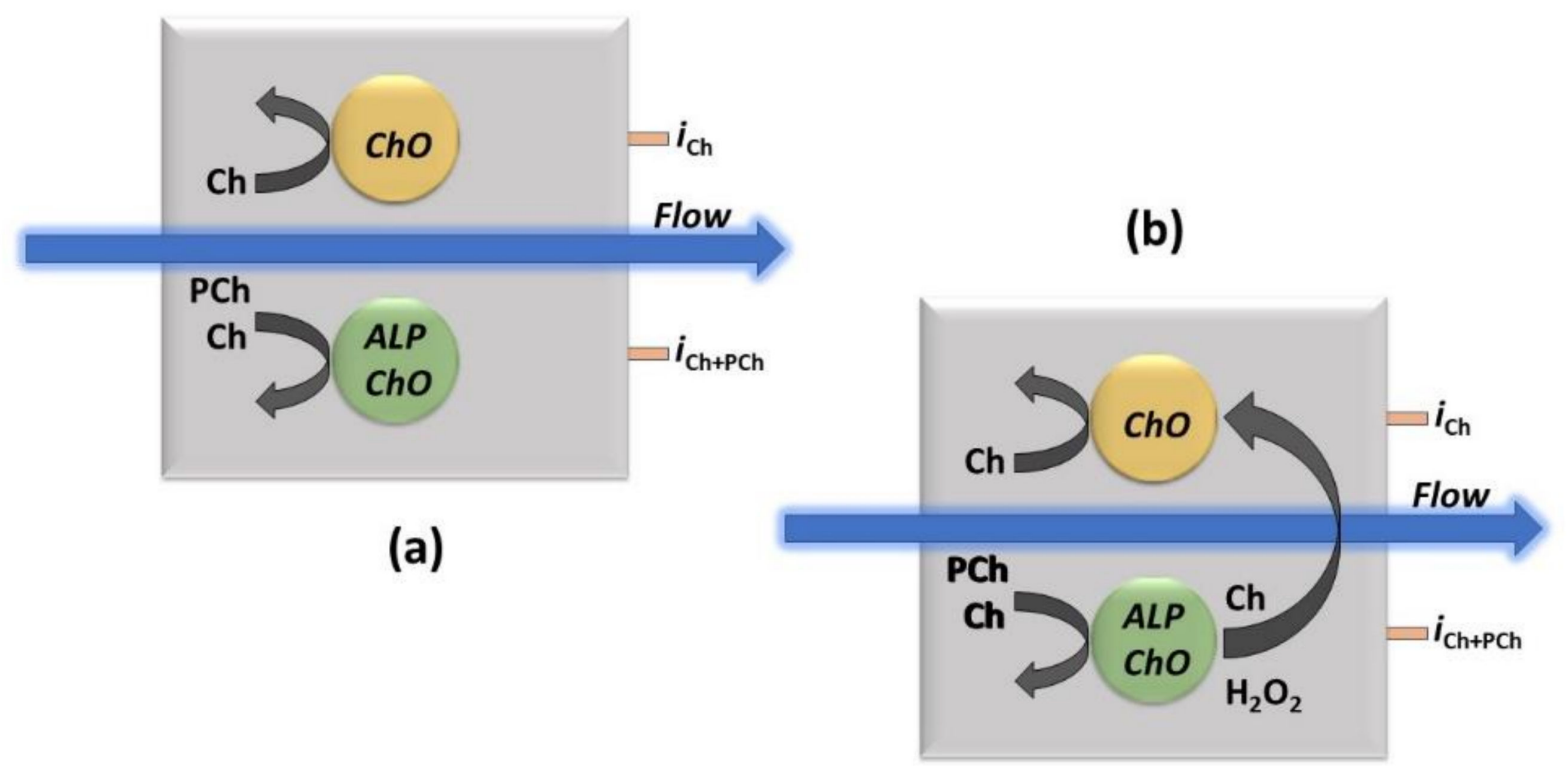
The approach for the simultaneous determination by the dual electrode (parallel configuration) amperometric biosensor thin-layer cell (as shown in Scheme S1 in Supplementary Materials) used in the flow injection experiments for Ch and PCh analysis (**a**) and the example of a crosstalk effect in measurements (**b**). ChO and ALP/ChO refer to ChO and ALP/ChO biosensors, respectively, the black curved arrows (left side in both sketches) represent the enzymatic detection, the blue ones, the carrier flow direction while *i*_Ch_ and *i*_PCh_ the currents for Ch and PCh measurements, respectively, as measured at their respective electrical connections. The larger black curved arrow (right side in right sketch) represents the potential crosstalk effect due to hydrogen peroxide detection from the side electrode.

Due to the enzymatic detection approach here used, where PCh needs firstly to be converted to Ch for its detection (see the enzyme reactions in the Introduction section), a single Pt/ChO-ALP biosensor can just perform an integrated determination of both analytes. The feasibility of a simultaneous determination of Ch and PCh by flow injection analysis has been here studied developing a dual electrode amperometric biosensor (see Scheme 1 and Scheme S1 in Supplementary Materials for further details), provided that the Pt/ChO-ALP biosensor gives additivity of Ch and PCh responses and crosstalk side effects due to e.g., hydrogen peroxide detection by both the electrodes are absent (vide infra).

**Figure 1:**
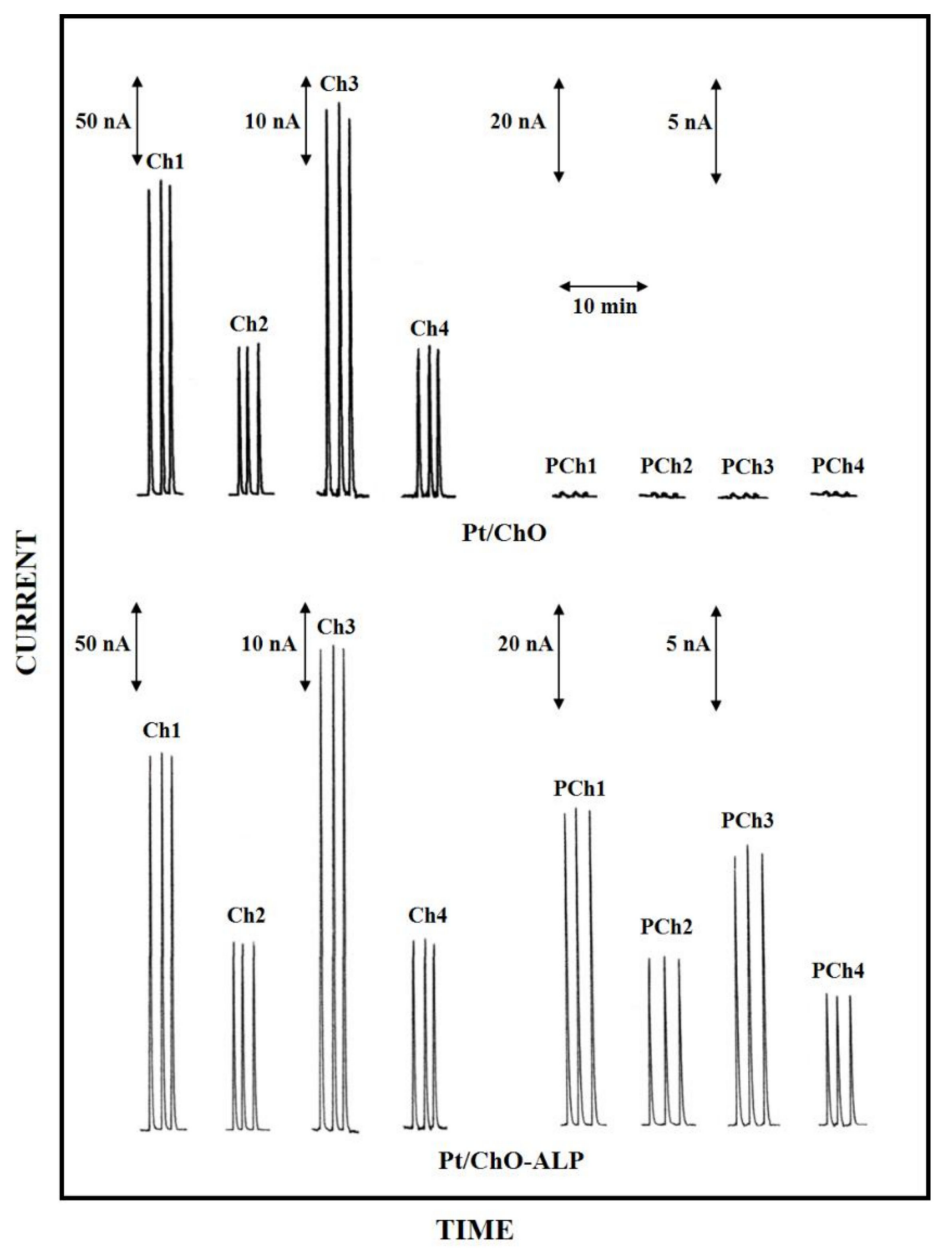
Typical flow injection responses at the dual electrode biosensor for replicate injections of Ch 0.1 mM (Ch1), 0.05 mM (Ch2), 0.025 mM (Ch3), 0.01 mM (Ch4) and PCh 0.5 mM (PCh1), 0.25 mM (PCh2), 0.1 mM (PCh3), 0.05 mM (PCh4). Upper and lower traces refer to responses at Pt/ChO and Pt/Cho-ALP biosensors, respectively. Carrier solution: borate buffer (pH 9, *I* 0.1 M); flow rate 1 mL/min; injection volume 20 µL. Other experimental conditions as described in Section 2.

In this respect, Figure 1 shows typical flow injection responses observed at the dual electrode, amperometric biosensor due to replicate injections of standard solutions of both analytes. Flow injection peaks showed peak shapes and widths quite similar to those observed for common electroactive substances (e.g., hydrogen peroxide) at bare Pt electrode, thus suggesting that the enzymatic conversion is much faster than flowing sample and the presence of the enzymatic membranes onto the electrode surface does not hinder the permeation of both substrates and the product of the enzymatic reaction.

Due to the detection scheme, Ch responses were observed at both the enzyme modified electrodes (i.e., Pt/ChO and Pt/ChO-ALP, left side upper and lower traces in Figure 1, respectively) while PCh responses were observed only at the Pt/ChO-ALP biosensor (right side lower traces in Figure 1), whereas they are practically absent at the Pt/ChO electrode (right side upper traces in Figure 1) if not due to injection artefacts (vide infra). As Figure 1 demonstrates, the dual electrode amperometric biosensors are practically crosstalk-free (i.e., no PCh responses were observed at the parallel Pt/ChO electrode due to e.g., hydrogen peroxide lateral diffusion from the side Pt/ChO-ALP biosensor). Further, the occurrence of somewhat cross-contamination from weakly bounded ALP onto ChO biosensor during their use (and thus generating crosstalk) was avoided by saturating any free GLU residues left onto the enzyme membrane by BSA (a provision used for biosensor preparation, see Section 2). Indeed, in some cases, a crosstalk effect was observed for some PCh batches, with spurious responses at the Pt/ChO biosensor, which increased with the PCh concentration increase: fortunately, this was only due to traces of Ch in some commercial preparations, as verified by purifying PCh standard solution by a strong cation exchange column, so care should be used in selecting and using PCh standards.

**Figure 2:**
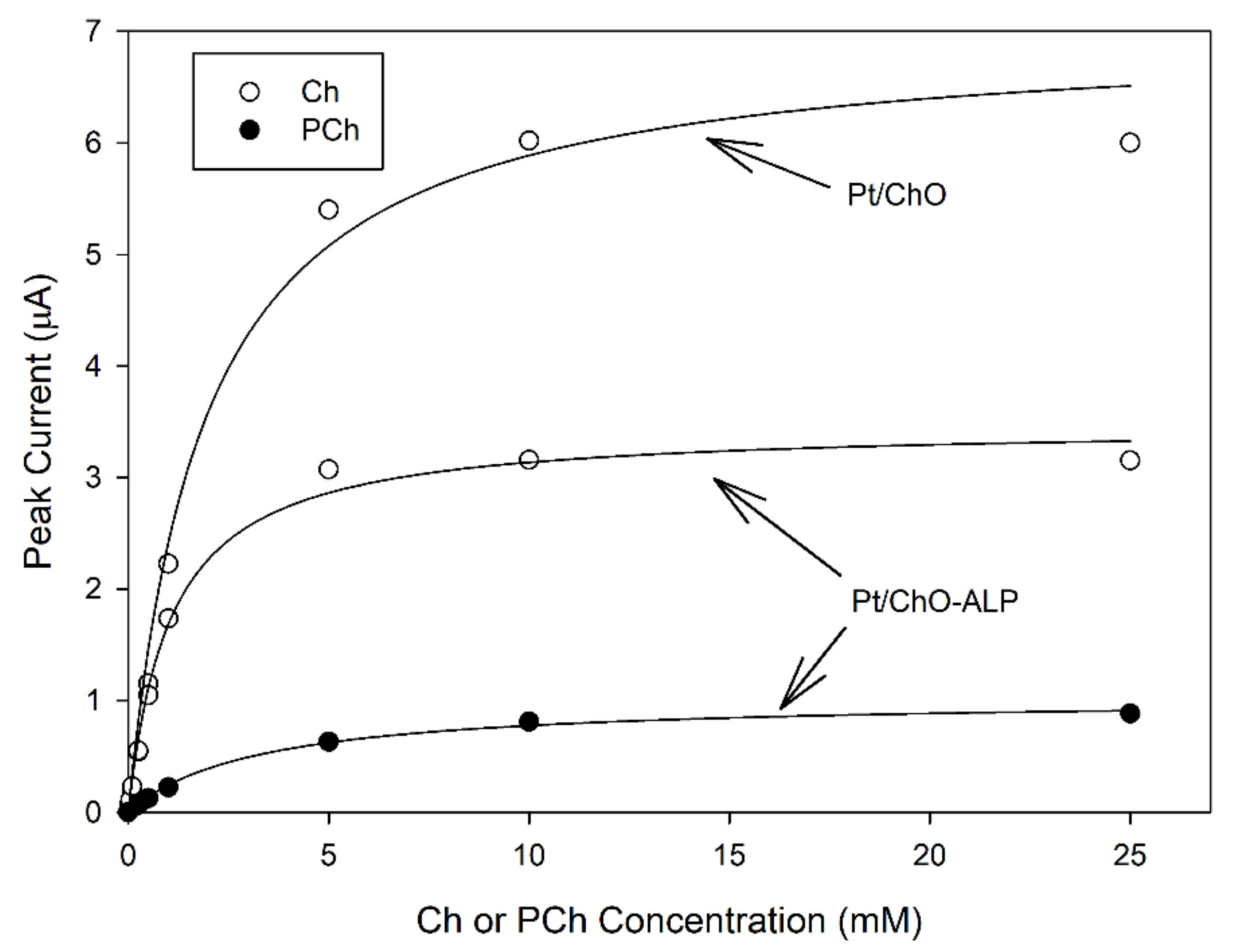
Calibration curves at the dual electrode biosensor for replicate injections of Ch and PCh. Continuous lines refer to Michaelis-Menten fitting of data. Carrier solution: borate buffer (pH 9, *I* 0.1 M); flow rate 1 mL/min; injection volume 20 µL. Other experimental conditions as described in the Section 2.

Figure 2 shows typical calibration curves at the dual electrode biosensor. As can be seen, all plots displayed linear and saturated responses at low and high concentrations, respectively, for both analytes and for both biosensors, confirming the kinetic behavior expected for an enzyme catalysis. Michaelis–Menten data fitting (correlation coefficients better than 0.997) gave apparent kinetic constants reported in Table 1. As Figure 2 and Table 1 show, ALP co-immobilization had a detrimental effect on ChO maximal response (i.e., the maximal current *I*’_max_ for Ch at Pt/ChO-ALP was about half with respect to Pt/ChO) but the apparent Michaelis–Menten constants *K*’_m_ were almost the same suggesting that the ChO loading in ChO-ALP membrane is lower, e.g., the co-immobilization of ALP lowers the efficiency in ChO immobilization. Finally, *I*’_max_ for PCh was even lower while *K*’_m_ was higher. Fortunately, all these kinetic features will not preclude the analytical performances of both biosensors, as it will show below.

Linear parts of calibration curves from Figure 1 are shown in Figure S2: as can be seen, the dual electrode biosensor gave excellent linear responses for both analytes and at both the electrodes. Linear fitting of their data (correlation coefficients better than 0.999) gave the sensitivities reported in Table 1: sensitivities towards Ch were practically identical for both the sensors and the responses were linear for more than 3 decades of Ch concentrations (see Table 1). Sensitivity towards PCh detection (see Table 1) was almost a tenth than Ch but sufficiently higher to permit PCh detection as expected in real sample (see below); of course, the lower sensitivity reduced the PCh linear range (see Table 1) due to a higher detection limit (see below). Lastly, the within-a-day coefficients of variations (CV) for replicate (*n* = 5) PCh injections were 4.3%, 2.1%, and 1.9% at 250, 25, and 5 µM PCh levels, respectively. At the same, the Pt/ChO-ALP electrode, CVs for replicate (*n* = 5) Ch injections were 2.9%, 2.3%, and 2.5% at 100, 10, and 1 µM PCh levels, respectively, whereas at the Pt/ChO electrode, were 1.3%, 3.0%, and 3.4% at the same Ch levels.

**Figure 3:**
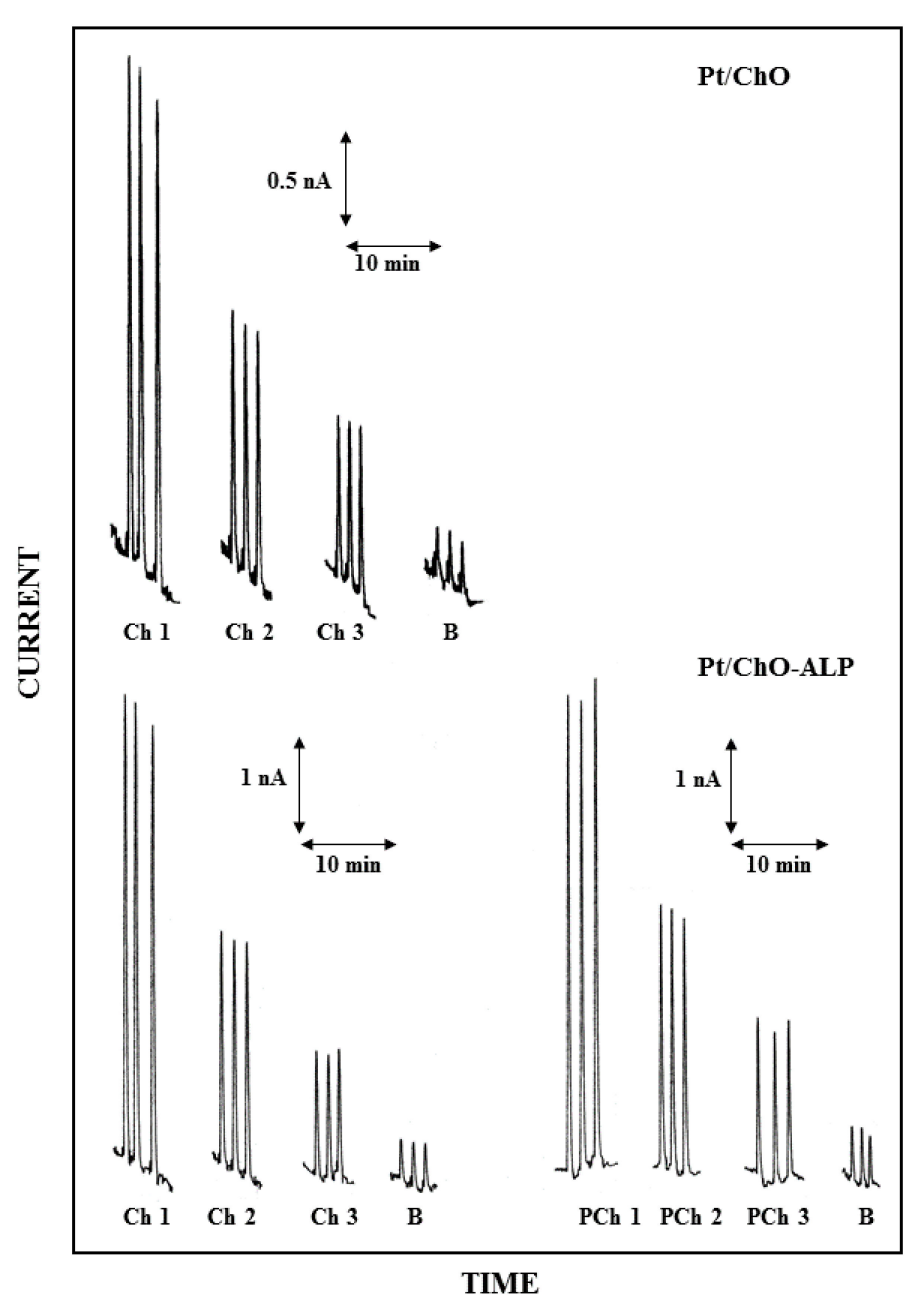
Typical flow injection responses at the dual electrode biosensor for replicate injections of Ch 2.5 µM (Ch1), 1.25 µM (Ch2), 0.625 µM (Ch3) and PCh 25 µM (PCh1), 12.5 µM (PCh2), 6.25 µM (PCh3); B refers to responses due to replicate injections of the carrier solution, i.e., the buffer used for preparing sample solutions. Upper and lower traces refer to responses at Pt/ChO and Pt/ChO-ALP biosensors, respectively. Carrier solution: borate buffer (pH 9, *I* 0.1 M); flow rate 1 mL/min; injection volume 20 µL. Other experimental conditions as described in Section 2.

Figure 3 shows the dual electrode biosensor responses for replicate injection of Ch and PCh at concentration levels close to their detection limits. As can be seen, the responses are mainly limited slightly by a signal drift (almost due to the peristaltic pump used in the flow injection setup) but significantly by the presence of injection artefacts on analyte determination (see responses in figure where B refers to replicate injections of the carrier solution) due to the change of pressure occurring at the injection step: both signal limitations were included in the noise floor analysis. In this respect, Table 1 reports the observed limit of detection (LOD) for Ch and PCh at a signal-to-noise ratio of 3. Please note that in Table 1 and whenever it applies, the absolute limit of detection expresses the minimum amount of analyte detectable, while the relative limit of detection refers to the minimum concentration of analyte detectable. Of course, these values can be significantly lowered by using a pulseless pumping system and an improved sample injector as proved elsewhere [38]. Nevertheless, the present LOD values are much better than those reported using the complex and expensive NMR spectroscopy techniques. In fact, for Ch, they are typically in the millimolar range [22,32], sometimes requiring scan times up to 10 h; only the high sophisticated 1H–14N HSQC detection technique [27] gives (estimated) LOD of about 2 µM using a 16 min scan time (reported LOD for Ch is 1 mM at a S/N = 1700), which is still higher than the present LODs. Analytical approaches using the enzimatic conversion, as in the present case, but using enzyme reactors and electrohemical detection, described higher LODs [34]. Further, neither LODs for PCh, neither a simultaneous determination with Ch were reported [34,35,36]. LC/MS standard approaches [14,15,17,18] stated in the best cases both absolute and relative LODs, comparable with those reported in Table 1; anyway, more complex and expensive LC/MS techniques [21] reported LODs down to nanomolar range. Even if not so sensitive as the last technique, the present approach shows linear ranges and LODs more than adequate for real sample analysis (see references [14,15,17,18,21,22,27,32,34,35,36] for sample and analyte ranges).

In flow injection approaches using an amperometric biosensor as in the present case, flow rate is well-known to rule the apparent enzyme kinetics and the measured currents [60]. In this respect, Figure S3 reports the flow rate dependences for Ch and PCh at the dual amperometric biosensor. For both analytes, the biosensor responses decreased, increasing the flow rate, leveling off starting from about 1 mL/min. This behavior, already reported and rationalized elsewhere (see for example references [37,38,39,42]), is anticipated since increasing the flow rate reduces the enzymatic conversion efficiency and/or diffuses away from the enzyme membrane (and hence from the Pt electrode) that produced hydrogen peroxide. Figure S3 apparently shows better analytical performances at low flow rate since of the higher responses: unfortunately, injection artefacts increased as well (see Figure S4), thus nullifying the increased response of analyte. Indeed, while at 0.2 mL/min, LOD for PCh was 6.5 µM (corresponding to 130 pmol injected), LODs for Ch at the same flow rate were 0.69 µM (13.7 pmol injected) and 0.78 µM (15.6 pmol injected) at Pt/ChO and Pt/ChO-ALP electrodes, respectively, i.e., comparable with those reported in Table 1. Accordingly, a flow rate of 1 mL/min was selected since the onset of the flow rate independence shown in Figure S3.

### 3.4. Simultaneous Determination of Ch and PCh at the Dual Electrode Biosensor

Even if the present dual electrode biosensor demonstrated excellent analytical performances for single analyte detection, a simultaneous determination of both analytes requires that all the molecular events (i.e., enzyme catalysis/electrochemical detection) occurring for Ch detection does not interfere with those similarly involved for PCh and vice versa. In the best case, the amperometric responses for both analytes should be linearly related to their concentration levels, in their respective linear ranges, as described by the following relations:

*I*_BI_ = K_1_ [Ch] + K_2_ [PCh] (1)

**Figure 4:**
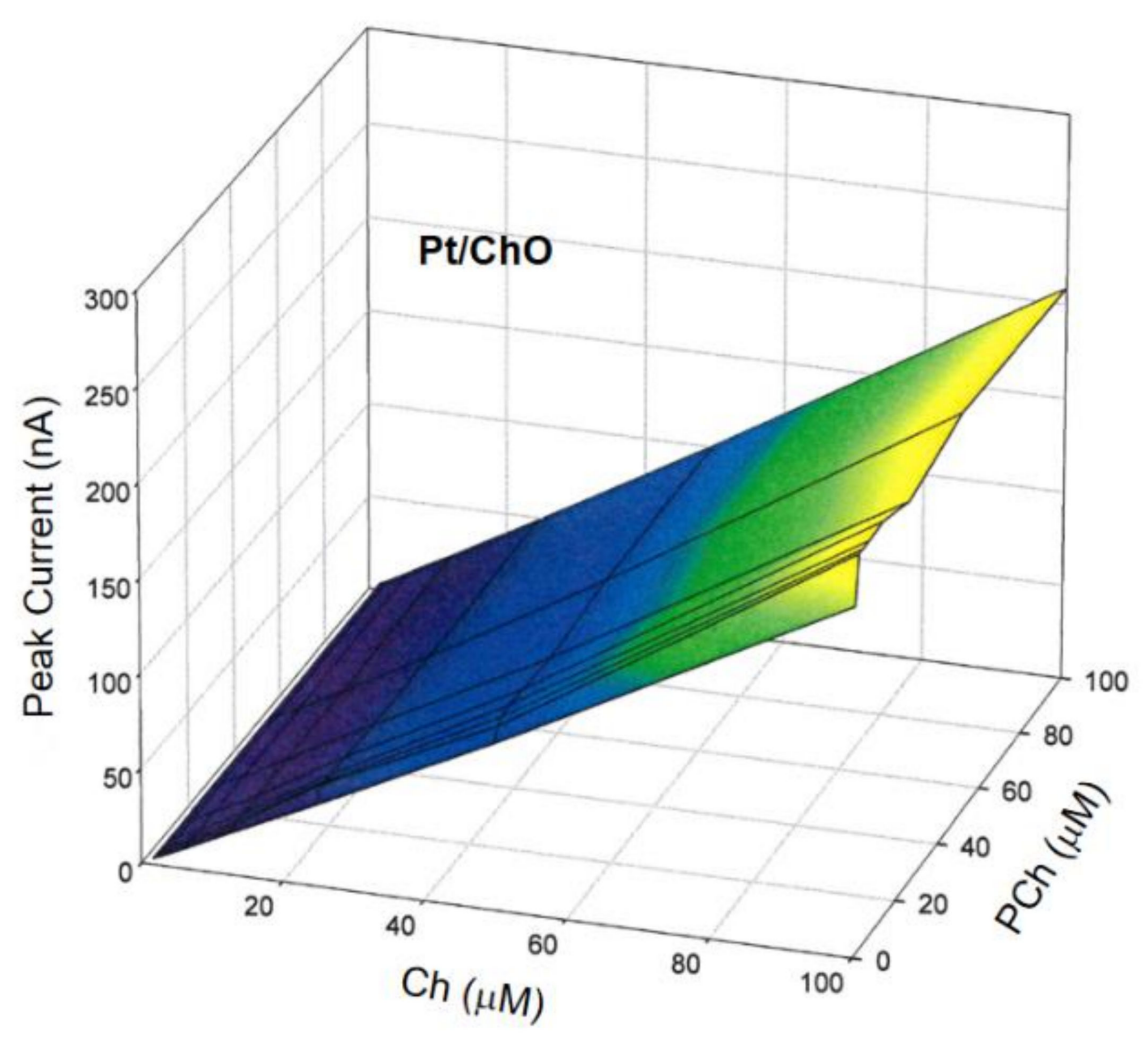
Calibration 3d plot at the Pt/ChO electrode of the dual electrode biosensor for replicate injections of mixtures of Ch and PCh. Carrier solution: borate buffer (pH 9, *I* 0.1 M); flow rate 1 mL/min; injection volume 20 µL. Other experimental conditions as described in Section 2.

**Figure 5:**
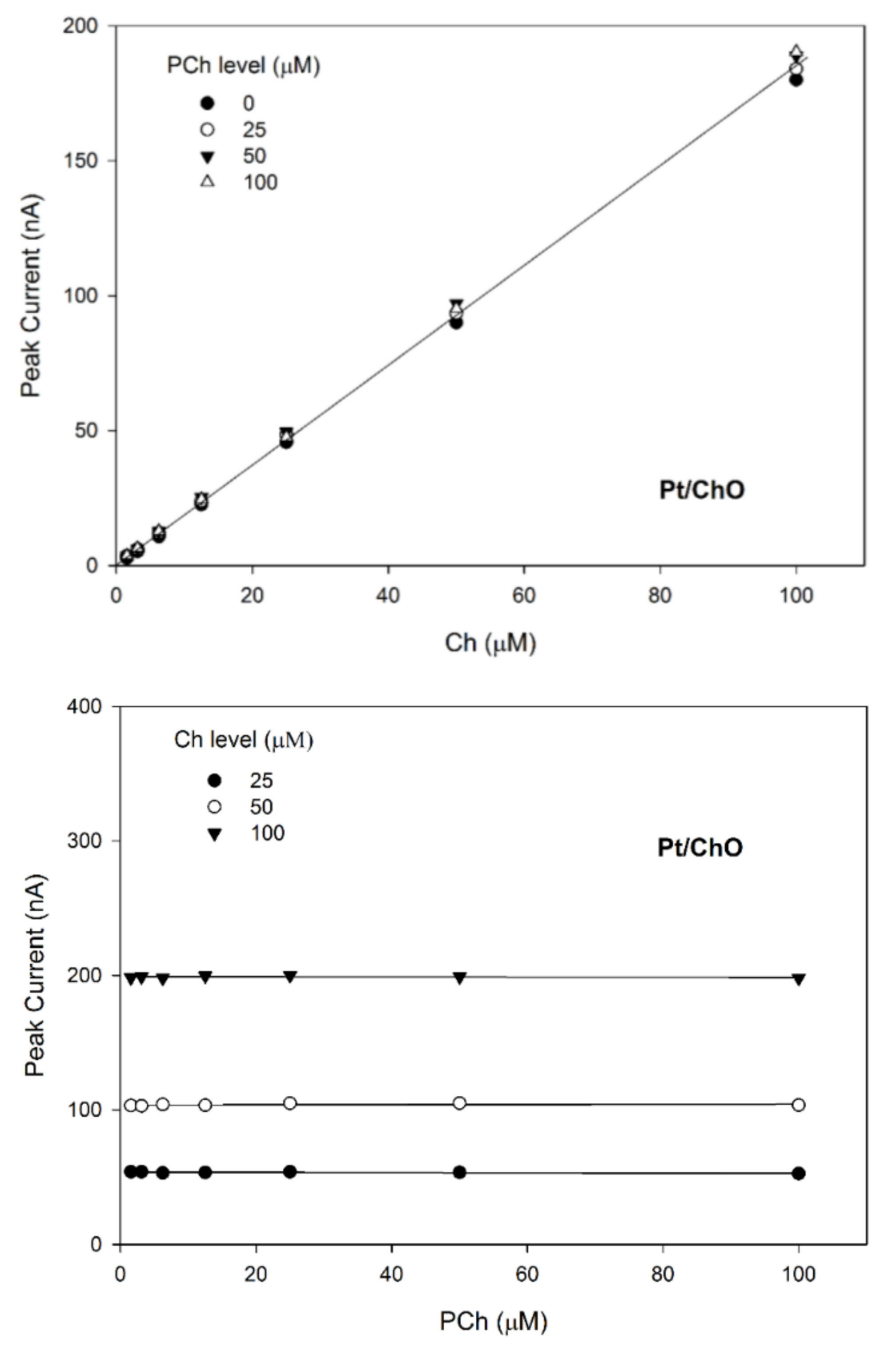
Calibration lines at the Pt/ChO electrode of the dual electrode biosensor for replicate injections of mixtures of Ch and PCh at several concentration levels of PCh (upper plot) and Ch (lower plot). Continuous lines refer to linear fitting of data. Carrier solution: borate buffer (pH 9, *I* 0.1 M); flow rate 1 mL/min; injection volume 20 µL. Other experimental conditions as described Section 2.

*I*_M_ = K_3_ [Ch] (2)

where *I*_BI_ and *I*_M_ are the amperometric responses at Pt/ChO-ALP and Pt/ChO electrodes, respectively, [Ch] and [PCh] are the concentration levels in the sample mixture of both analytes, while K_1_, K_2_, and K_3_ are the respective analyte sensitivities. To validate the additivity of analyte responses, several Ch and PCh mixtures were analyzed using the present dual electrode biosensor for a concentration range as listed in Table 1 in the linear range column. In this respect, Figure 4 shows the 3d plot of the observed responses at the Pt/ChO electrode, while Figure 5 shows some relevant slices of the 3d plot at some constant analyte levels.

Due to the specificity towards Ch and the lack of any crosstalk effects, the amperometric responses shown in Figure 4 for Pt/ChO of the present dual electrode biosensor are linearly related to Ch but independent of PCh. Accordingly, the detector response is represented by a plane with a slope equal to Ch sensitivity at the Pt/ChO electrode, parallel to the PCh concentration axis and with zero intercept. Particularly, Figure 5 (upper plot) shows the linear dependence of responses towards Ch whatever the PCh level in the sample mixture while the constant behaviors observed in the lower plots in Figure 5 certified the independence of Ch responses from PCh whatever the Ch level. Accordingly, the responses of the dual electrode biosensor at the Pt/ChO detector can be described by Equation (2).

**Figure 6:**
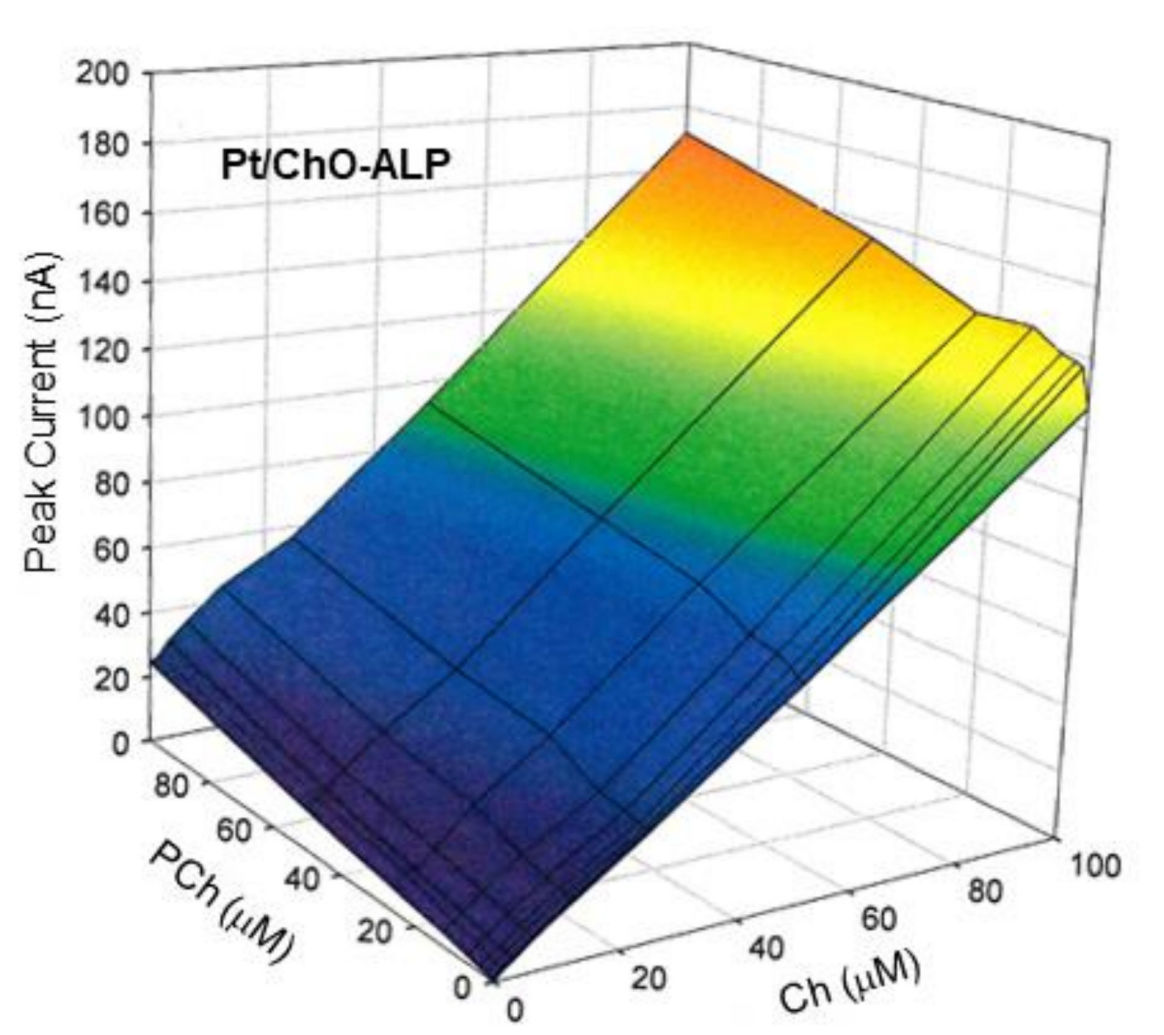
Calibration 3d plot at the Pt/ChO-ALP electrode of the dual electrode biosensor for replicate injections of mixtures of Ch and PCh. Carrier solution: borate buffer (pH 9, *I* 0.1 M); flow rate 1 mL/min; injection volume 20 µL. Other experimental conditions as described in Section 2.

**Figure 7:**
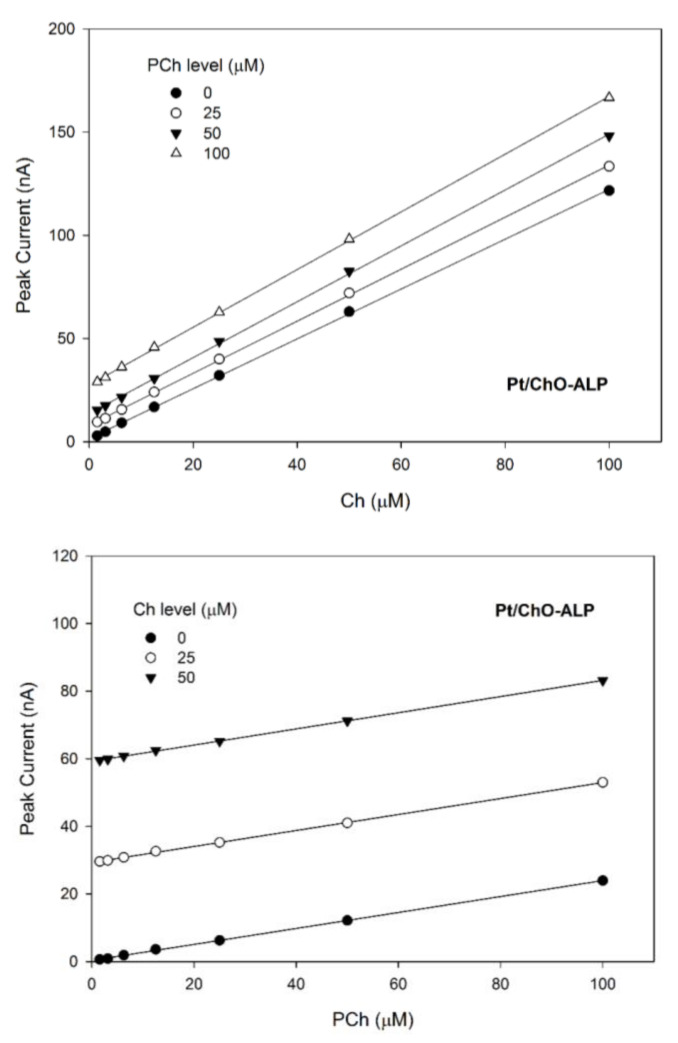
Calibration lines at the Pt/ChO-ALP electrode of the dual electrode biosensor for replicate injections of mixtures of Ch and PCh at several concentration levels of PCh (upper plot) and Ch (lower plot). Continuous lines refer to linear fitting of data. Carrier solution: borate buffer (pH 9, *I* 0.1 M); flow rate 1 mL/min; injection volume 20 µL. Other experimental conditions as described in Section 2.

On the other hand, Figure 6 shows the 3d plot of the observed responses at the Pt/ChO-ALP electrode, while Figure 7 shows some relevant slices of the 3d plot at some constant analyte levels. As Figure 6 shows, the amperometric responses at the Pt/ChO-ALP electrode are linearly and additively dependent from both analytes: now the plane representing the detector response has its lower vertex at zero and tilted as the sensitivities toward Ch and PCh. Particularly, both plots in Figure 7 confirmed the additivity of responses for both analytes in the relevant linear ranges reported in Table 1. According to these experimental findings, Equation (1) can be used to describe the responses of Ch and PCh at the Pt/ChO-ALP electrode of the present biosensor; in particular, this relation can be simplified to:

*I*_BI_ = K_1_ [Ch] + A_2_ (3)

for Ch determination (Figure 7, upper plot), and to:

*I*_BI_ = A_1 +_ K_2_ [PCh] (4)

for PCh determination (Figure 7, lower plot), A_1_ and A_2_ representing the constant values of Ch and PCh, respectively.

Due to these experimental results, proper calibration of the Pt/ChO electrode (i.e., the determination of K_3_ in Equation (2)) can permit to analyze Ch levels in the sample whatever the PCh concentration. Similarly, calibration of the Pt/ChO-ALP electrode toward Ch and PCh (i.e., the determination of K_1_ and K_2_ in Equation (1)) can allow PCh determination in Ch/PCh mixtures since the Ch contribution to the overall response can be calculated by the simultaneous response at the Pt/ChO electrode.

Finally, it should be noted that the behavior of the dual electrode biosensors has been studied even for Ch and PCh concentrations greater than their respective linear range upper limits, i.e., when the enzyme kinetics of both enzymes are not linear and saturating (see Figure 2 and Table 1 for the relevant analyte levels). This characterization, of course, had no relevance for the analytical studies of the proposed biosensor due to the non-linear responses so that was not here described. Nevertheless, a significant depression of Ch responses was observed for high PCh levels, thus suggesting ChO inhibition by PCh, an inhibition phenomenon never reported for this enzyme. A detailed study about this behavior is reported elsewhere.

### 3.5. Interference-Free Performances of the Dual Electrode Biosensor

Even if the enzymatic transduction assures specific biorecognition of Ch and PCh, unfortunately the succeeding electrochemical detection is poorly selective since any endogenous electroactive compounds in the sample could give an amperometric response if oxidable at the potential of hydrogen peroxide detection. Of course, this side effect at least biases the analyte responses. As a proof of this deleterious effect, Figure S5 compares typical flow injection responses of Ch and PCh at the dual electrode biosensor with those due to representative endogenous electroactive compounds, namely ascorbate (AA), urate (AU), and cysteine (CYS) at their respective physiological levels in biological fluids. As can be seen, the co-occurrence of these compounds generates significant bias on analyte response, even many times higher than e.g., the Ch response (compare with AU responses). Even if lowering the flow rate is expected to increase the biosensor response (see above) while decreasing the faradic currents due to interferent oxidation, the bias introduced in the analyte measurement is still dramatic even at low flow rate (as Figure S6 shows), so an application to real sample analysis appears impracticable using this biosensor configuration.

The usual approach in amperometric biosensors to overcomes this adverse effect is the use of discrete membranes (like polycarbonate, cellulose, and so) onto the electrode surface, selectively limiting the diffusion of endogenous electroactive compounds present in the sample. In addition to be scarcely effective, the use of these relatively macroscopic and thick membranes limits also, usually by distortion, the analyte response and precludes any miniaturization of the sensing device [48,49,50,51,52]. Electropolymerized non-conducting films with built-in permselectivity [39,41,42,43,44,45,46,47,48,49,50,51,52,53,54,61] have extensively demonstrated to protect the sensor surface from electrode fouling effects (e.g., due to high molecular weight proteins in sample) and to remove, with unmatched efficiency, common electroactive interfering species in glucose, Ch, and Ach, as well as in lysine determination in real sample analysis (e.g., blood, serum, dialysates, tissue homogenate). In this approach, a suitable monomer (e.g., o-aminophenol, pyrrole, 2-naphthol) is electrooxidized at the Pt electrode surface so forming in situ a polymeric, non-conducting, strong adherent film with adverse permselectivity towards many endogenous electroactive compounds; apart from minimizing these undesired, interferent effects, this approach easily permits the deposition of very thin-film (so not distorting the analyte response), even with multilayer facilities and, due to the electrochemical deposition technique there used, permits the miniaturization of any sensing device.

**Figure 8:**
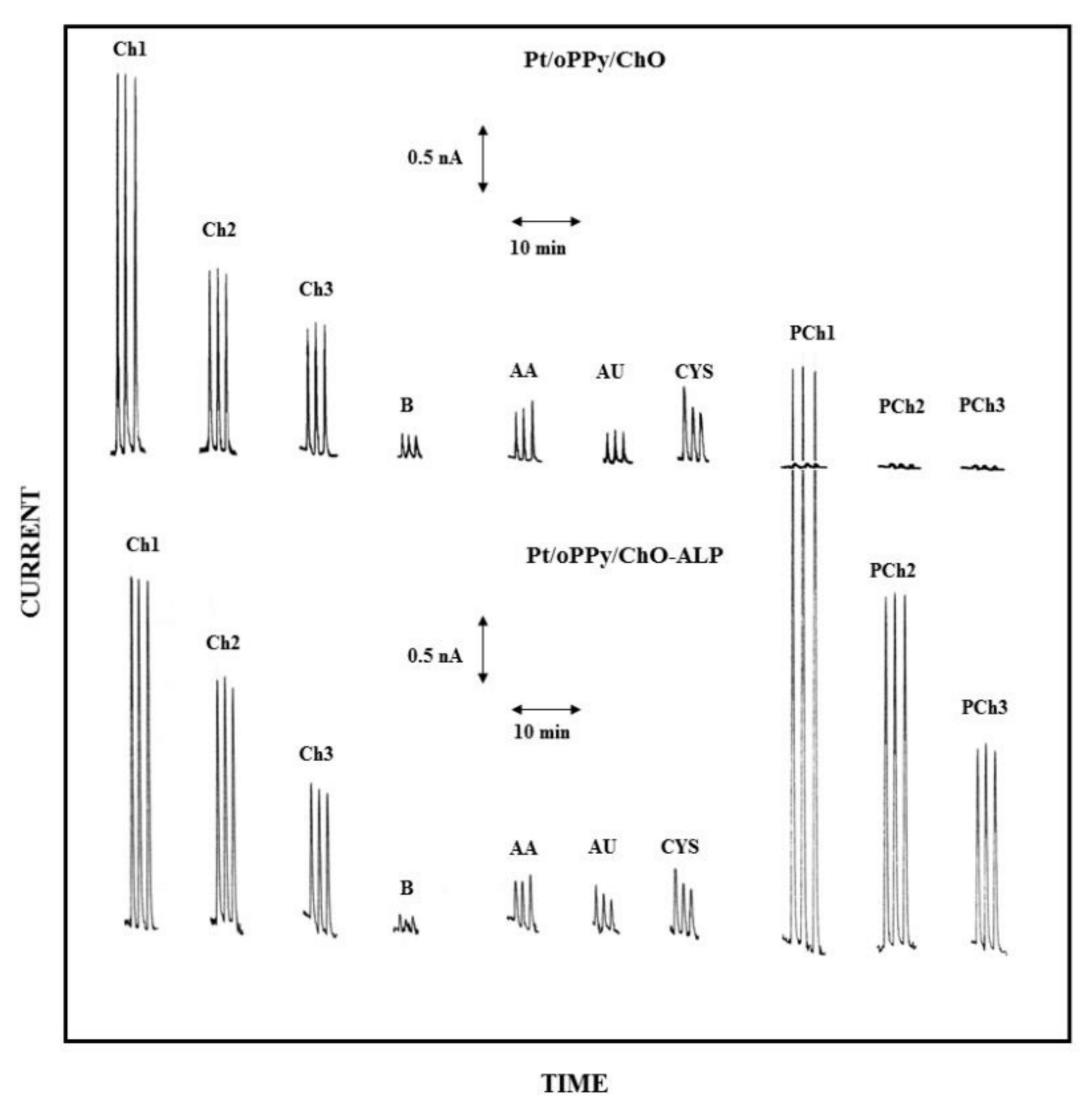
Typical flow injection responses at the oPPy modified dual electrode biosensor for replicate injections of Ch 2.5 µM (Ch1), 1.25 µM (Ch2), 0.625 µM (Ch3), PCh 25 µM (PCh1), 12.5 µM (PCh2), 6.25 µM (PCh3) compared to responses due to AA 0.1 mM, AU 0.5 mM, and CYS 0.2 mM; B refers to responses due to replicate injections of the carrier solution, i.e., the buffer used for preparing sample solutions. Upper and lower traces refer to responses at Pt/oPPy/ChO and Pt/oPPy/ChO-ALP biosensors, respectively. Carrier solution: borate buffer (pH 9, *I* 0.1 M); flow rate 1 mL/min; injection volume 20 µL. Other experimental conditions as described in Section 2.

Accordingly, in the present case, both the electrodes of the dual electrode biosensor were modified, before enzyme immobilization, by filming the bare Pt electrodes with overoxidized polypyrrole (oPPy): the resulting electrodes, here described as Pt/oPPy/ChO and Pt/oPPy/ChO-ALP electrodes, proved highly stable in stirred or flowing solution since modified with a thin film quite adherent to the enzyme membrane and to the underneath Pt surface. The high selectivity of the electrochemical detection obtained here by this approach is shown by Figure 8, where low Ch and PCh levels are compared to responses due to some endogenous electroactive compounds at their physiological concentrations; as the figure demonstrates, the modification with oPPy dramatically reduces the interferent effect due to these substances (compare Figure 8 with Figure S5 where this electrode modification was not there used): in particular, the interference effect due to AA and CYS is practically suppressed whereas the interference signal due to AU is even much more suppressed. Similar interference-free performances were also observed at low flow rate (e.g., 0.2 mL/min, see Figure S7). Notably, also the undesired artefacts due to sample injection were significantly reduced (compare responses B in Figure 8 with those already shown in Figure 3): undoubtedly, the oPPy modification makes somewhat the electrode immunes from the dielectric constant changes due to the injected buffer and/or pressure changes during sample injection. At the same time, the presence of oPPy film onto the Pt electrode does not limit or reduce the analytical signal, i.e., the oxidation of the enzymatic produced hydrogen peroxide, as a comparison of Figure 8 with Figure 3 (where oPPy modification is absent) shows. In this respect, Table 2 (and Table S2 for low flow rates) summarizes the analytical performances of the dual electrode biosensor modified by oPPy: with respect to data already reported in Table 1, sensitivities were just a bit lower that the unmodified case while linear ranges were practically identical; on the contrary, absolute and relative LODs were significantly lower as a consequence of the reduced noise (e.g., injection artefacts) in measurements, confirming that the performances of the biosensor were quite adequate for real sample analysis even at low analyte levels.

A quantitative mode to describe the interference effect due to endogenous electroactive compounds relies on the use of the analyte bias factor, i.e., the concentration (or the amount) of the analyte (Ch or PCh in the present case) which generates a response equal to those of interferent species at their maximal physiological levels. Accordingly, Table 3 reports the tested bias values on Ch and PCh measurements as due to AA, AU, and CYS interferences. As can be seen, in the worst cases, the interferent effects due to the investigated interferents mimic an analyte response (bias) corresponding to 0.25 and 1.6 µM for Ch and PCh, respectively. All the observed biases were comparable if not lower than LODs performed for Ch and PCh: in particular, the biases introduced in analyte measurements were comparable to the observed artefacts due to injection so noise due sample analysis cannot be distinguished from interference response. Of course, this assured that the present biosensor, as modified by oPPy, is certainly specific towards Ch and PCh at least for the endogenous interference compounds here tested.

The analytical performances of the here described dual electrode biosensor demonstrated quite appealing in terms of sensitivities, linear ranges, LODs, and high specificity so application to real sample analysis appears promising: unfortunately, biological sample like biological fluids and tissues, where significant Ch and PCh levels are expected (see the Introduction section), were not available, particularly during these last times. Anyway, the proposed dual electrode biosensor was tested for the simultaneous determination of Ch and PCh in biologically-like synthetic sample (i.e., bovine serum albumin solutions enriched with endogenous electroactive compounds) spiked with known amount of several Ch/PCh mixtures: according to a paired Student’s *t*-test (1—α = 0.95 level), the found analyte levels were not significantly different from those observed for Ch/PCh standards so excluding any matrix effects in analyte quantitation due to high molecular proteins in the sample. Further, the successfully application of alike biosensors in high complex sample like foods and biological fluids [38,40,41,42,43,44,45,48,51,52,53,54] could comfort about a successfully application even for the more complex sample.

### 3.6. Stability of the Dual Electrode Biosensor

The stability of the biosensor was studied either at short or long time. The operational stability was tested by repetitive injection of Ch/PCh mixtures under the continuous run of the flow injection system: during all the working day (8—12 h), no significant difference in responses was observed for both the analyte and the within-a-day CVs were those already here reported (see above).

Storage and long-term stability of the dual electrode biosensor was studied by discontinuously monitoring the Ch and PCh responses of a biosensor continuously used over about one month and stored in buffer at 4 °C in the dark when not in use: no significant loss in sensitivity was observed during more than 30 days, after an initial sensitivity drop of about 30% observed gradually during the first 5 days.

## 4. Conclusions

Ch and PCh levels in tissues are associated with tissue growth and so to carcinogenesis. Very few methods have been reported till now demonstrating capable of Ch or PCh analysis and even fewer of a simultaneous determination of both analytes, while the ratio of Ch/PCh levels, rather than their absolute levels, is useful as a diagnostic and prognostic marker in these pathologies. Since all these methods rely on highly sophisticated and expensive analytical techniques (NMR spectroscopy or GC/LC-high resolution mass spectrometry), there is the necessity to design and develop more simple, fast, specific, and accurate analytical approaches, as usually necessary in clinical and medical fields.

This paper demonstrates that biosensing could permit the development of simple, easy to fabricate, and easy to use analytical devices, satisfying the above goals. By co-crosslinking of ChO or ChO-ALP enzymes onto the Pt surface of a simple electrochemical thin-layer cell, a novel PCh biosensor has been developed, a biosensor never reported before. Further, coupling the developed biosensor with a parallel ChO sensor in a dual electrode electrochemical cell, a crosstalk-free dual electrode biosensor has been also developed, permitting the simultaneous determination of Ch and PCh in flow injection analysis. This novel sensing device performed remarkably in terms of sensitivity, linear range, and limit of detection, thus exceeding in most cases the more complex analytical instrumentations.

To circumvent matrix effects in real sample analysis due to high molecular weight proteins and endogenous electroactive compounds generating deleterious fouling effects onto the electrodes and unpredictable bias on analyte responses, respectively, electropolymerized non-conducting films with built-in permselectivity have been used to increase the selectivity of the electrochemical detection. Thus, electrode modification by oPPy permitted the development a fouling- and interferent-free dual electrode biosensor which appeared surely promising for the simultaneous determination of Ch and PCh in a real sample like biological fluids and tissues. Work in this direction is in progress in the present laboratory.

## Figures and Tables

**Table 1:** Analytical performances at the (unmodified) dual electrode biosensor ^1^.

Analyte	Biosensor	*K*’_m_^2^ (mM)	*I*’_max_^3^ (µA)	Sensitivity (µA/mM)	Linear Range (µM)	LOD ^4^ (µM/pmol)
PCh	Pt/ChO-ALP	3.6 ± 0.2	1.03 ± 0.02	0.216	7–1000	6.9/139
Ch	Pt/ChO-ALP	1.19 ± 0.07	3.6 ± 0.1	2.03	0.7–1000	0.68/13.6
Ch	Pt/ChO	2.1 ± 0.3	7.3 ± 0.3	2.13	0.7–1000	0.65/13.0

^1^ Carrier solution: borate buffer (pH 9, *I* 0.1 M); flow rate 1 mL/min; injection volume 20 µL. Other experimental conditions as described in Section 2; ^2^ *K*’_m_: apparent Michaelis-Menten constant; ^3^ *I*’_max_: apparent maximal current; ^4^ LOD: relative (µM) and absolute (pmol injected) limit of detection calculated at a signal-to-noise ratio of 3.

**Table 2:** Analytical performances at the oPPy modified dual electrode biosensor ^1^.

Analyte	Biosensor	Sensitivity (µA/mM)	Linear Range (µM)	LOD ^2^ (µM/pmol)
PCh	Pt/oPPy/ChO-ALP	0.197	3–1000	2.7/53.6
Ch	Pt/oPPy/ChO-ALP	1.30	0.3–1000	0.19/3.9
Ch	Pt/oPPy/ChO	1.39	0.3–1000	0.28/5.6

^1^ Carrier solution: borate buffer (pH 9, *I* 0.1 M); flow rate 1 mL/min; injection volume 20 µL. Other experimental conditions as described in Section 2. ^2^ LOD: relative (µM) and absolute (pmol injected) limit of detection calculated at a signal-to-noise ratio of 3.

**Table 3:** Biases on Ch and PCh measurements by interferents at the oPPy modified dual electrode biosensor ^1^.

Analyte	Biosensor	Bias (µM) For:		
		AA (0.1 mM)	AU (0.5 mM)	CYS (0.2 mM)
PCh	Pt/oPPy/ChO-ALP	1.5	1.2	1.6
Ch	Pt/oPPy/ChO-ALP	0.19	0.15	0.22
Ch	Pt/oPPy/ChO	0.2	0.14	0.25

^1^ Carrier solution: borate buffer (pH 9, *I* 0.1 M); flow rate 1 mL/min; injection volume 20 µL. Other experimental conditions as described in Section 2.

## Data Availability

Not applicable.

## Supplementary Materials

The following are available online at https://www.mdpi.com/article/10.3390/s21103545/s1, Scheme S1. Schematic diagram of the flow injection setup used in the experiments showing in particular the electrochemical cell and the relevant dual electrode biosensor used in the amperometric measurements (the reference electrode and the flow cell gaskets were omitted for sake of figure clarity). Table S1. Comparison of main analytical performances of choline oxidase (ChO)/alkaline phosphatase (ALP) biosensors produced with different ALP sources. Figure S1. Normalized sensitivities towards Ch and PCh *vs* pH at a typical rotating disk Pt/ChO-ALP electrode. Supporting electrolyte: acetate/borate/tris buffer (*I* 0.1 M). Rotation rate: 1000 rpm. Experimental condition as described in the Materials and Methods section of the paper. Figure S2. Linear parts of the calibration curves at the dual electrode biosensor for replicate injections of Ch and PCh as shown in Figure 1 (see the relevant section of the paper). Continuous lines refer to linear fitting of data (correlation coefficients better than 0.999). Carrier solution: borate buffer (pH 9, *I* 0.1 M); flow rate 1 mL/min; injection volume 20 µL. Other experimental conditions as described in the Materials and Methods section of the paper. Figure S3. Normalized peak currents vs flow rate plots for replicate injections of Ch (2.5 µM) and PCh (25 µM) at Pt/ChO (left) and Pt/ChO-ALP (right) dual electrode biosensor. Carrier solution: borate buffer (pH 9, I 0.1 M); injection volume 20 µL. Other experimental conditions as described in the Materials and Methods section of the paper. Figure S4. Typical flow injection responses at the dual electrode biosensor for replicate injections of Ch 2.5 µM (Ch1), 1.25 µM (Ch2), 0.625 µM (Ch3) and PCh 25 µM (PCh1), 12.5 µM (PCh2), 6.25 µM (PCh3); B refers to responses due to replicate injections of the carrier solution, i.e., the buffer used for preparing sample solutions. Upper and lower traces refer to responses at Pt/ChO and Pt/Cho-ALP biosensors, respectively. Carrier solution: borate buffer (pH 9, I 0.1 M); flow rate 0.2 mL/min; injection volume 20 µL. Other experimental conditions as described in the Materials and Methods section of the paper. Figure S5. Comparison of flow injection responses at the dual electrode biosensor for replicate injections of Ascorbate (AA, 0.1 mM), Urate (AU, 0.5 mM), Cysteine (CYS, 0.2 mM), Ch (0.1 mM), and PCh (1 mM) at a flow rate of 1 mL/min. Upper and lower traces refer to responses at Pt/ChO and Pt/Cho-ALP biosensors, respectively. Carrier solution: borate buffer (pH 9, I 0.1 M); injection volume 20 µL. Other experimental conditions as described in the Materials and Methods section of the paper. Figure S6. Comparison of flow injection responses at the dual electrode biosensor for replicate injections of Ascorbate (AA, 0.1 mM), Urate (AU, 0.5 mM), Cysteine (CYS, 0.2 mM), Ch (0.1 mM), and PCh (1 mM) at a flow rate of 0.2 mL/min. Upper and lower traces refer to responses at Pt/ChO and Pt/Cho-ALP biosensors, respectively. Carrier solution: borate buffer (pH 9, I 0.1 M); injection volume 20 µL. Other experimental conditions as described in the Materials and Methods section of the paper. Figure S7. Typical flow injection responses at the oPPy modified dual electrode biosensor for replicate injections of Ch 2.5 µM (Ch1), 1.25 µM (Ch2), 0.625 µM (Ch3), PCh 25 µM (PCh1), 12.5 µM (PCh2), 6.25 µM (PCh3) compared to responses due to AA 0.1 mM, AU 0.5 mM, and CYS 0.2 mM; B refers to responses due to replicate injections of the carrier solution, i.e., the buffer used for preparing sample solutions. Upper and lower traces refer to responses at Pt/oPPy/ChO and Pt/oPPy/ChO-ALP biosensors, respectively. Carrier solution: borate buffer (pH 9, I 0.1 M); flow rate 0.2 mL/min; injection volume 20 µL. Other experimental conditions as described in the Materials and Methods section of the paper. Table S2. Analytical performances at the oPPy modified dual electrode biosensor^1^ at low flow rate.
