# Supplementary material for: A Crosstalk- and Interferent-Free Dual Electrode Amperometric Biosensor for the Simultaneous Determination of Choline and Phosphocholine
Source: Sensors (Basel). 2021 May 19;21(10):3545. doi: 10.3390/s21103545 (PMC8160789; doi:10.3390/s21103545)
Supplement: Supplementary file 1 [file sensors-21-03545-s001.zip › sensors-1229722-supplementary.pdf]

sensors

Supplementary Materials

A Crosstalk- and Interferent-Free Dual Electrode Amperometric Biosensor for the Simultaneous Determination of Choline and Phosphocholine

Rosanna Ciriello and Antonio Guerrieri *

Dipartimento di Scienze, Università degli Studi della Basilicata, Viale dell'Ateneo Lucano 10, 85100 Potenza, Italy; rosanna.ciriello@unibas.it
* Correspondence: antonio.guerrieri@unibas.it; Tel.: +39-0971-205460

Citation: Ciriello, R.; Guerrieri, A. A Crosstalk- and Interferent-Free Dual Electrode Amperometric Biosensor for the Simultaneous Determination of Choline and Phosphocholine. *Sensors* **2021**, *21*, 3545. https://doi.org/10.3390/s21103545

Academic Editor: Ajeet Kaushik

Received: 5 May 2021
Accepted: 14 May 2021
Published: 19 May 2021

Publisher's Note: MDPI stays neutral with regard to jurisdictional claims in published maps and institutional affiliations.

Copyright: © 2021 by the authors. Submitted for possible open access publication under the terms and conditions of the Creative Commons Attribution (CC BY) license (http://creativecommons.org/licenses/by/4.0/).

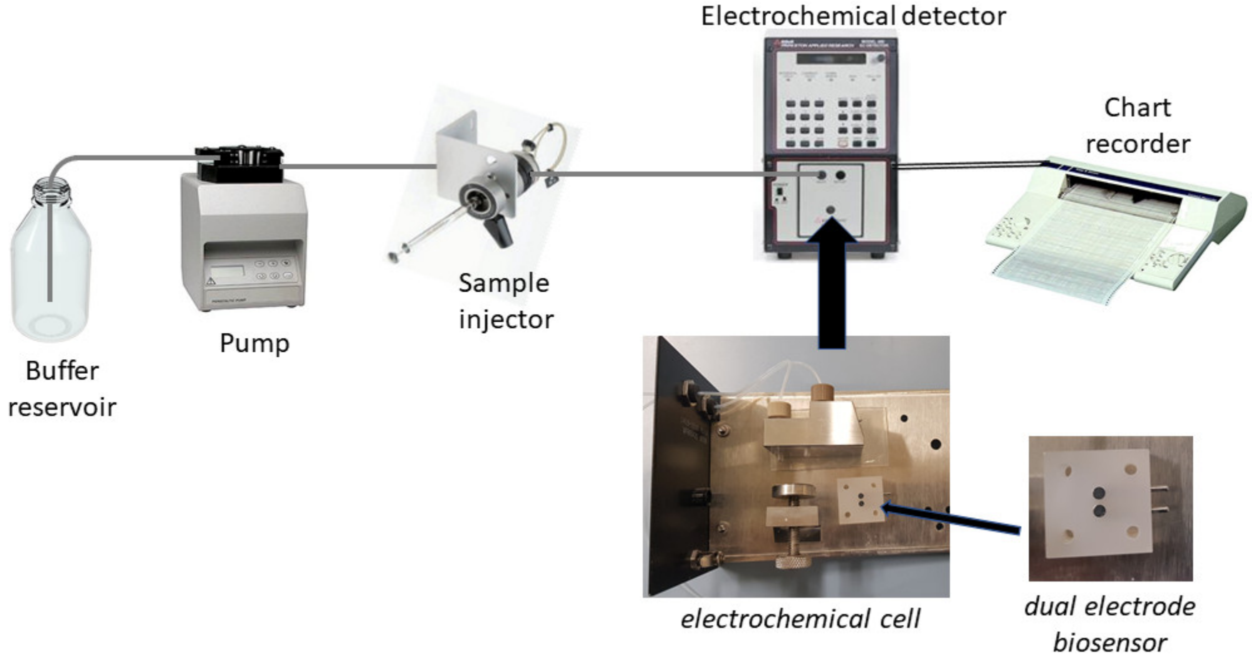

Scheme S1. Schematic diagram of the flow injection setup used in the experiments showing in particular the electrochemical cell and the relevant dual electrode biosensor used in the amperometric measurements (the reference electrode and the flow cell gaskets were omitted for sake of figure clarity).

Table S1. Comparison of main analytical performances of choline oxidase (ChO)/alkaline phosphatase (ALP) biosensors produced with different ALP sources.

Enzyme Source	Units[1]	Enzyme Code[2]	Main Analytical Performances of Biosensors
Bovine intestinal mucosa	13 DEA units/mg	P7640	Low PCh[3] sensitivity, low PCh/Ch[4] sensitivity ratio, high response time
Bovine calf intestinal	2,000 DEA units	P7923	Acceptable PCh sensitivity, low PCh/Ch sensitivity ratio, high response time, very low long-term stability
Shrimp	1,300 DEA units	P9088	Limited PCh/Ch sensitivity ratio, very high response time
Bovine intestinal mucosa	22,990 U/mL	79385	High PCh sensitivity, satisfactory PCh/Ch sensitivity ratio, low response time, high operational and long-term stability

Biosensor preparation and experimental condition as described in Section 2 of the paper. [1]One DEA unit is defined as the amount of protein that hydrolyzes 1 μmol of 4-nitrophenyl phosphate per minute at pH 9.8 and 37 °C. [2]Commercial code: see Section 2 of the paper for relevant details and commercial source. [3]phosphocholine. [4]choline.

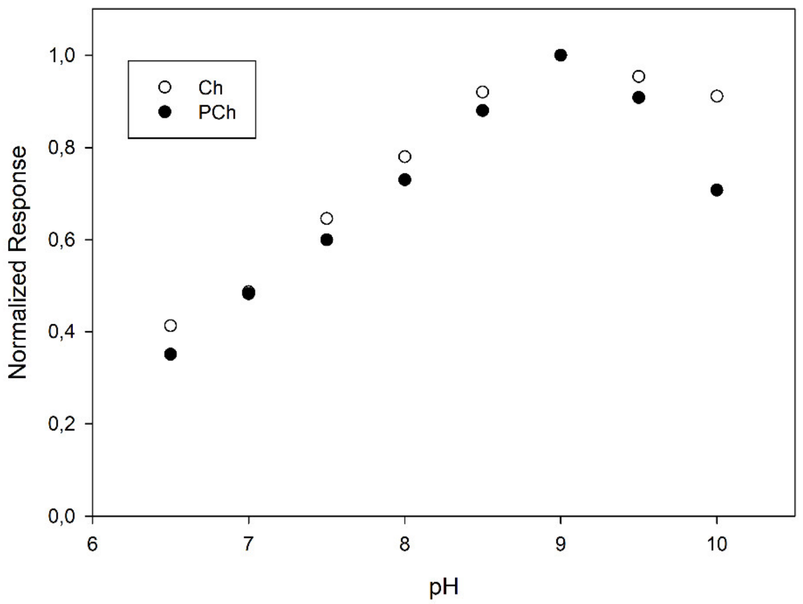

Figure S1. Normalized sensitivities towards Ch and PCh *vs* pH at a typical rotating disk Pt/ChO-ALP electrode. Supporting electrolyte: acetate/borate/tris buffer (*I* 0.1 M). Rotation rate: 1000 rpm. Experimental condition as described in Section 2 of the paper.

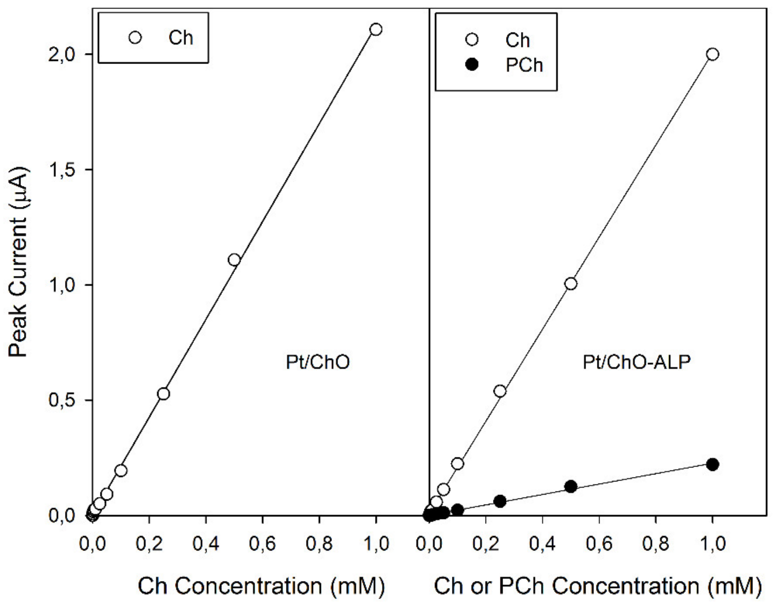

Figure S2. Linear parts of the calibration curves at the dual electrode biosensor for replicate injections of Ch and PCh as shown in Figure 1 (see the relevant section of the paper). Continuous lines refer to linear fitting of data (correlation coefficients better than 0.999). Carrier solution: borate buffer (pH 9, *I* 0.1M); flow rate 1 mL/min; injection volume 20 μL. Other experimental conditions as described in Section 2 of the paper.

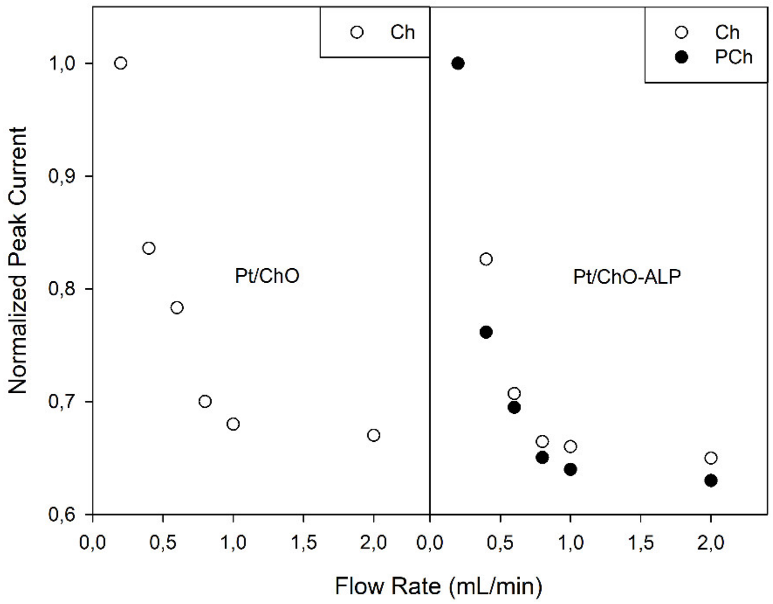

Figure S3. Normalized peak currents *vs* flow rate plots for replicate injections of Ch (2.5 μM) and PCh (25 μM) at Pt/ChO (left) and Pt/ChO-ALP (right) dual electrode biosensor. Carrier solution: borate buffer (pH 9, *I* 0.1M); injection volume 20 μL. Other experimental conditions as described in Section 2 of the paper.

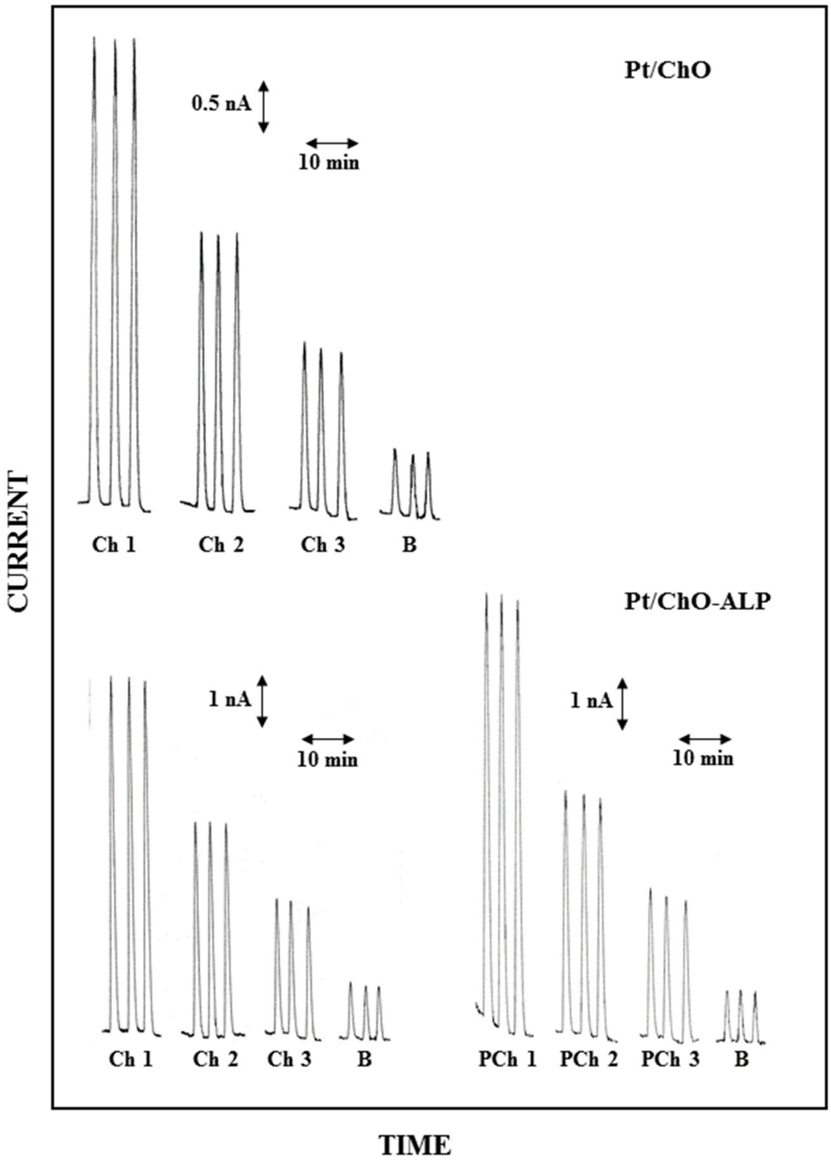

Figure S4. Typical flow injection responses at the dual electrode biosensor for replicate injections of Ch 2.5 µM (Ch1), 1.25 µM (Ch2), 0.625 µM (Ch3) and PCh 25 µM (PCh1), 12.5 µM (PCh2), 6.25 µM (PCh3); B refers to responses due to replicate injections of the carrier solution, i.e. the buffer used for preparing sample solutions. Upper and lower traces refer to responses at Pt/ChO and Pt/Cho-ALP biosensors, respectively. Carrier solution: borate buffer (pH 9, *I* 0.1M); flow rate 0.2 mL/min; injection volume 20 µL. Other experimental conditions as described in Section 2 of the paper.

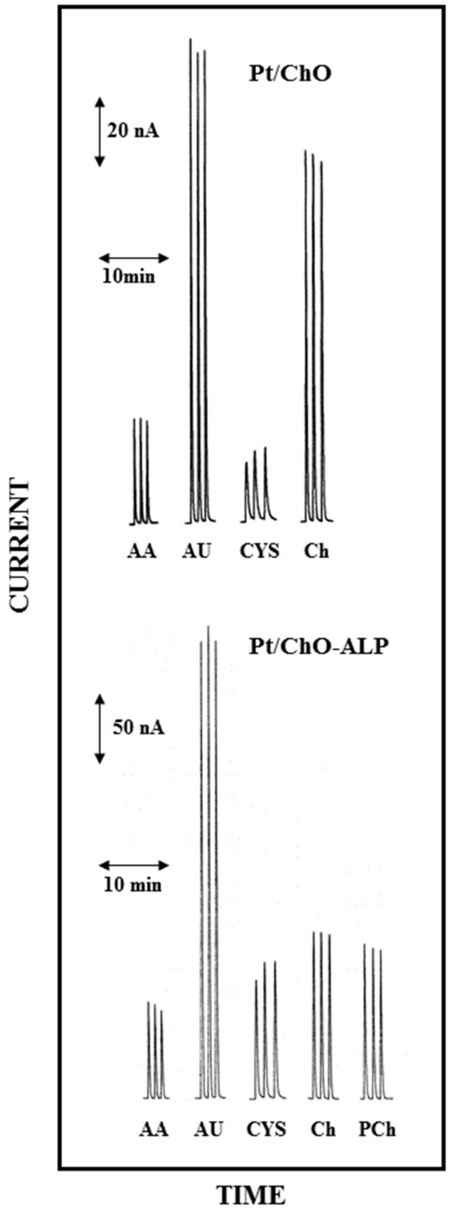

Figure S5. Comparison of flow injection responses at the dual electrode biosensor for replicate injections of Ascorbate (AA, 0.1 mM), Urate (AU, 0.5 mM) Cysteine (CYS, 0.2 mM) , Ch (0.1 mM) and PCh (1 mM) at a flow rate of 1 mL/min. Upper and lower traces refer to responses at Pt/ChO and Pt/Cho-ALP biosensors, respectively. Carrier solution: borate buffer (pH 9, *I* 0.1M); injection volume 20 μL. Other experimental conditions as described in Section 2 of the paper.

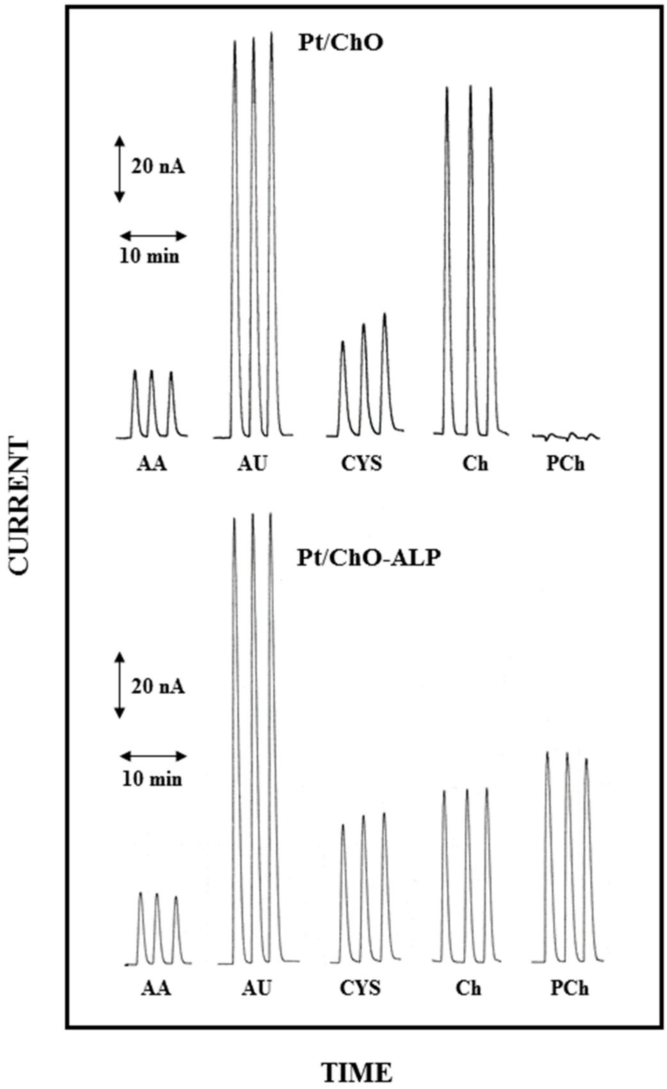

Figure 6. Comparison of flow injection responses at the dual electrode biosensor for replicate injections of Ascorbate (AA, 0.1 mM), Urate (AU, 0.5 mM) Cysteine (CYS, 0.2 mM) , Ch (0.1 mM) and PCh (1 mM) at a flow rate of 0.2 mL/min. Upper and lower traces refer to responses at Pt/ChO and Pt/Cho-ALP biosensors, respectively. Carrier solution: borate buffer (pH 9, *I* 0.1M); injection volume 20 μL. Other experimental conditions as described in Section 2 of the paper.

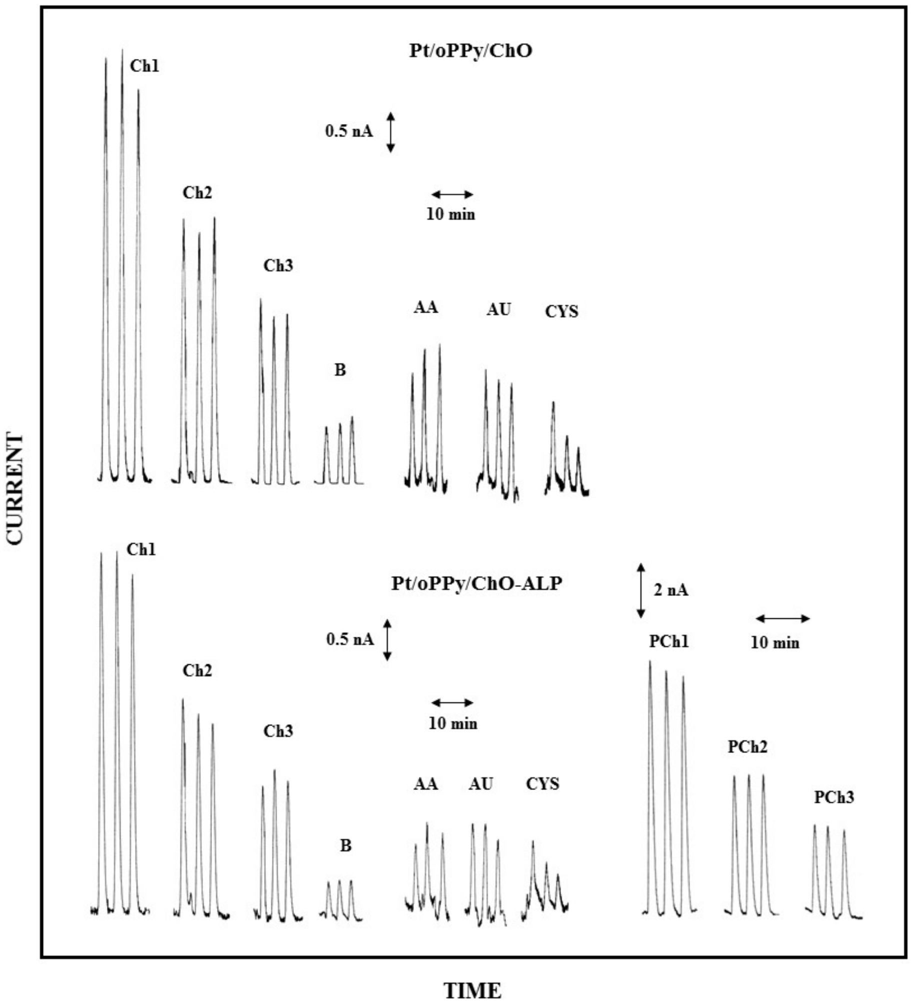

Figure S7. Typical flow injection responses at the oPPy modified dual electrode biosensor for replicate injections of Ch 2.5 μM (Ch1), 1.25 μM (Ch2), 0.625 μM (Ch3), PCh 25 μM (PCh1), 12.5 μM (PCh2), 6.25 μM (PCh3) compared to responses due to AA 0.1 mM, AU 0.5 mM and CYS 0.2 mM; B refers to responses due to replicate injections of the carrier solution, i.e. the buffer used for preparing sample solutions. Upper and lower traces refer to responses at Pt/oPPy/ChO and Pt/oPPy/ChO-ALP biosensors, respectively. Carrier solution: borate buffer (pH 9, *I* 0.1M); flow rate 0.2 mL/min; injection volume 20 μL. Other experimental conditions as described in Section 2 of the paper.

Table 2. Analytical performances at the oPPy modified dual electrode biosensor[1] at low flow rate.

Analyte	Biosensor	Sensitivity (μA/mM)	Linear Range (μM)	LOD[2] (μM/pmol)
PCh	Pt/oPPy/ChO-ALP	0.318	8–1,000	8.4/169
Ch	Pt/oPPy/ChO-ALP	1.64	0.7–1,000	0.57/11.4
Ch	Pt/oPPy/ChO	1.89	0.7–1,000	0.64/12.8

[1]Carrier solution: borate buffer (pH 9, *I* 0.1M); flow rate 0.2 mL/min; injection volume 20 μL. Other experimental conditions as described in Section 2. [2]LOD: relative (μM) and absolute (pmol injected) limit of detection calculated at a signal-to-noise ratio of 3.
